# Establishing relationships between particle-induced in vitro and in vivo inflammation endpoints to better extrapolate between in vitro markers and in vivo fibrosis

**DOI:** 10.1186/s12989-023-00516-y

**Published:** 2023-02-09

**Authors:** Polly McLean, William Mueller, Ilse Gosens, Flemming R. Cassee, Barbara Rothen-Rutishauser, Matthew Boyles, Lang Tran

**Affiliations:** 1 Institute of Occupational Medicine (IOM), Edinburgh, UK; 2 National Institute for Public Health and the Environment – RIVM, Bilthoven, The Netherlands; 3 Institute for Risk Assessment Sciences, Utrecht University, Utrecht, The Netherlands; 4 Adolphe Merkle Institute, University of Fribourg, Chemin des Verdiers 4, 1700 Fribourg, Switzerland

## Abstract

**Background:**

Toxicity assessment for regulatory purposes is starting to move away from traditional in vivo methods and towards new approach methodologies (NAM) such as high-throughput in vitro models and computational tools. For materials with limited hazard information, utilising quantitative Adverse Outcome Pathways (AOPs) in a testing strategy involving NAM can produce information relevant for risk assessment. The aim of this work was to determine the feasibility of linking in vitro endpoints to in vivo events, and moreover to key events associated with the onset of a chosen adverse outcome to aid in the development of NAM testing strategies. To do this, we focussed on the adverse outcome pathway (AOP) relating to the onset of pulmonary fibrosis.

**Results:**

We extracted in vivo and in vitro dose–response information for particles known to induce this pulmonary fibrosis (crystalline silica, specifically α-quartz). To test the in vivo–in vitro extrapolation (IVIVE) determined for crystalline silica, cerium dioxide nanoparticles (nano-CeO_2_) were used as a case study allowing us to evaluate our findings with a less studied substance. The IVIVE methodology outlined in this paper is formed of five steps, which can be more generally summarised into two categories (i) aligning the in vivo and in vitro dosimetry, (ii) comparing the dose–response curves and derivation of conversion factors.

**Conclusion:**

Our analysis shows promising results with regards to correlation of in vitro cytokine secretion to in vivo acute pulmonary inflammation assessed by polymorphonuclear leukocyte influx, most notable is the potential of using IL-6 and IL-1β cytokine secretion from simple in vitro submerged models as a screening tool to assess the likelihood of lung inflammation at an early stage in product development, hence allowing a more targeted investigation using either a smaller, more targeted in vivo study or in the future a more complex in vitro protocol. This paper also highlights the strengths and limitations as well as the current difficulties in performing IVIVE assessment and suggestions for overcoming these issues.

**Supplementary Information:**

The online version contains supplementary material available at 10.1186/s12989-023-00516-y.

## Introduction

Traditional chemical risk assessment methodology relies on the use of animal models, which is now discouraged, not only due to ethical reasons but also from concerns over the predictivity of animal data for human responses [1–3]. In order to replace traditional animal assessment, alternative testing strategies are needed with improved quality, efficiency and speed of chemical hazard and risk assessments [4]. The UK National Research Council (NRC) have published various reports [5–7], the first of which was released in 2007, outlining their vision and strategy with regards to modern toxicity testing and primarily focussed on new approach methodologies (NAM) such as high-throughput in vitro models and computational approaches as the best ways to replace these traditional animal models. This modern view on toxicity testing is of particular interest to novel materials that are being developed at a fast rate but in low quantities, and therefore do not yet meet requirements for regulatory assessment. High-throughput methods for assessing the risk of novel materials are preferred as these not only reduce the cost for the producers but also allow a large number of materials to be tested at once, with results obtained far quicker compared to animal methods.

For chemicals with well-described physico-chemical information, effective hazard ranking can be achieved by modelling the data obtained from high-throughput screening methods, as has been done by the US Environmental Protection Agency (EPA) with their ToxCast database [8]. However, the chemicals contained within the ToxCast Screening Library are largely pesticides, and no such information is available for other categories such as (novel) engineered materials.

For materials with limited hazard information, utilising quantitative Adverse Outcome Pathways (AOPs) in an in vitro testing strategy can produce information relevant for risk assessment [9]. AOPs can be a useful tool to determine the possible in vivo adverse outcome (AO) by investigating the effect of an inhalable particle with regards to various key events (KEs), such as cellular inflammatory responses and cytotoxicity. At present, there are no AOPs yet that also consider at what dose level the next (key) event is initiated, and are therefore mainly applied to investigate which marker for a key event should be included in NAMs. This information may be used to structure a targeted in vivo testing strategy to reduce the number of animals required to gain informed results. However, once well-established, it is hoped that these AOPs can be used to provide insights on in vitro–in vivo extrapolation (IVIVE) that would allow only in vitro assays to be required for chemical risk assessment, including the calculation of DNEL (Derived No-Effect Levels) values [10].

Others have described a procedure to extrapolate in vitro doses to human exposure levels using a combined in vitro–in vivo dosimetry method for the endpoint of (acute) lung inflammation utilising titanium dioxide as a case study [11]. Recently, Ma-Hock and colleagues proposed an IVIVE six-step procedure: (1) determine in vivo exposure; (2) identify in vivo organ burden at lowest observed adverse effect concentration; (3) extrapolate in vivo organ burden to in vitro effective dose; (4) extrapolate in vitro effective dose to nominal concentration; (5) set dose ranges to establish dose–response relationships; and (6) consider uncertainties and specificities of in vitro test system [12].

The approach detailed in this paper focusses on IVIVE, similar to that outlined by Ma-Hock et al*.* [12], which aims to take dose–response information from well-established in vivo responses (related to an in vivo AO) and correlate these with endpoints measured from in vitro assays (relevant to KE for this AO). This process can test if investigating particular KEs in vitro is sufficient to infer the onset of the chosen AO. Of course, once relationships have been identified between in vivo and in vitro endpoints, this extrapolation can go both ways, helping to enable effective correlation to human responses for risk assessment purposes. This IVIVE approach is already successfully utilised in the field of pharmacology, whereby human daily exposure levels can be calculated relevant to the in vitro test concentrations [13–16]. This is possible for chemicals (or families of chemicals) with well-defined human toxicity pathways such as endocrine disruptors, and is facilitated by knowledge on defined human dose as is possible in pharmacology. For occupational exposure to chemicals, human dose metrics via inhalation are less well defined. Therefore, our approach adapts the animal to human extrapolation already utilised by regulators [10, 17] to include a preceding extrapolation from in vitro models.

**Fig. 1:**
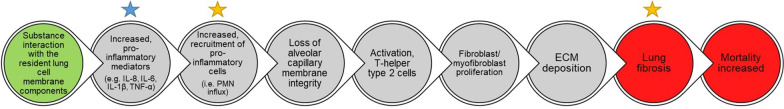
Schematic of AOP 173. AOP 173, “Substance interaction with the lung resident cell membrane components leading to lung fibrosis” adapted from [20]. Green box identifies the molecular initiating event (MIE), grey boxes are key events (KEs) and red boxes show adverse outcomes (AO). Yellow stars identify the focus of our in vivo literature search and the blue star identifies the KE of importance for our in vitro literature search

For the IVIVE approach used here, we have selected lung fibrosis as our target adverse outcome, given the evidence of particle-induced lung fibrosis upon inhalation in response to numerous particles and fibres [18, 19], and provides an opportunity to assess numerous stages and various KE [9] (Fig. 1). This development heavily relies upon on the available data, and as such, given its data-richness within published literature and databases, crystalline silica (α-quartz) was selected as our test material for proof-of-concept with inflammatory and fibrotic properties; with nano-CeO_2_ used as a case study to supplement our findings with a less studied substance. It is envisaged that if successful, this IVIVE can be used with engineered nanomaterials (ENMs) to test the likelihood of inhalation exposure resulting in inflammation-derived lung fibrosis, and to what extent this might occur compared to the well-described behaviour of α-quartz. This testing hypothesis will enable faster, more efficient hazard assessment to assist producers in following a safe-by-design approach.

## General approach

The methodology outlined in this report was used in the first instance to develop an IVIVE model for inflammation-induced lung fibrosis following exposure to α-quartz. This model scenario was chosen as there is a well-known mode of action for this particle toxicity [9] and it is known to occur in both humans and rats [21], therefore comparison of in vitro data to rats in vivo is relevant for human hazard of lung fibrosis. This is particularly useful as in vitro models are typically developed using human cells. To facilitate this comparison, available data from relevant literature was used as the key tool for IVIVE. Data on in vivo inflammation by means of polymorphonuclear leukocyte (PMN) influx, was compared to numerous in vitro markers that can describe inflammation in vivo. Using substantial literature sourcing, screening and data extraction, numerous in vivo and in vitro endpoints were compared and evaluated for suitability, which primarily led to comparisons made between the increased recruitment of pro-inflammatory cells (an early KE from the AOP for lung fibrosis Fig. 1) in vivo, and (pro-)inflammatory cytokine secretion in vitro, as has also been defined by Halappanavar et al*.* [3] as most suitable to assess inflammogenic potential of particles. Where successful comparisons were made, we investigated the possibility of deriving conversion factors applicable for extrapolation facilitating IVIVE.

**Fig. 2:**
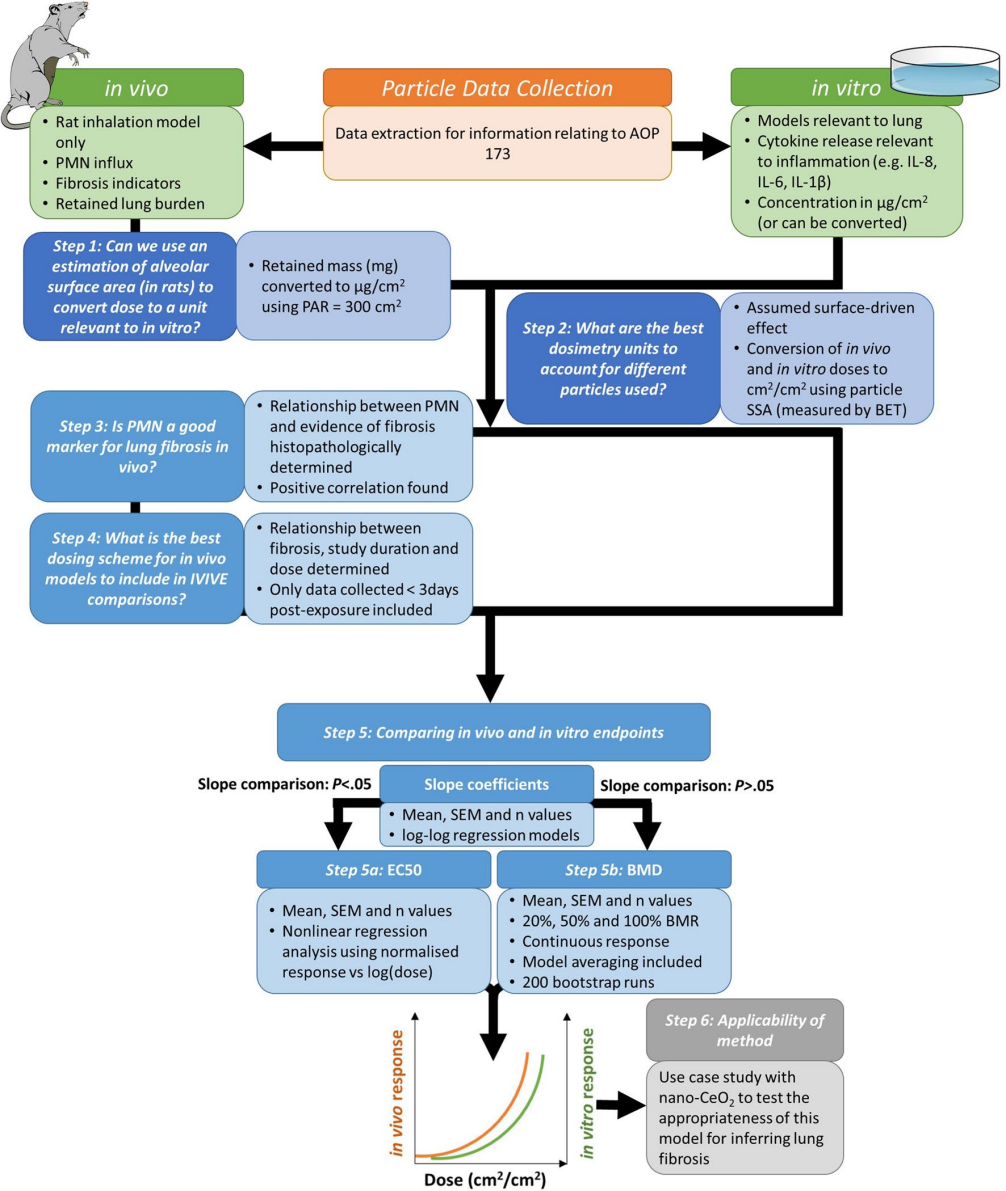
Schematic of the IVIVE procedure followed. Our approach included initial literature search and data extraction, followed by a six-step procedure to determine the feasibility of extrapolating between in vitro markers and in vivo fibrosis. First we aligned the in vivo and in vitro dosimetry by converting in vivo dosimetry to mass per surface area of tissue (Step 1) and then converted the mass per surface area dosimetry to units that reflect the surface-driven effect (i.e. cm^2^/cm^2^, Step 2). Next we compared endpoints by first determining if there is a correlation between PMN influx and fibrosis in vivo (Step 3), investigating the relationship between fibrosis and in vivo study protocols (Step 4) and then comparing PMN influx in vivo with cytotoxicity and inflammatory endpoints in vitro (Step 5). Step 5 was further split into two possible approaches, the first (Step 5a) involved dose comparisons at a central point of the curve e.g. by EC50 analysis, when in vivo and in vitro responses were aligned (*P* < .05). A second approach (Step 5b), which in theory can be applied to any curve shape, used a point-of-departure (PoD) analysis of the curves using BMD analysis. Finally, we studied the applicability of the method for inferring lung fibrosis from promising cytokine/chemokine endpoints in vitro using nano-CeO_2_ as a case study material

The analysis steps we have followed is provided in Fig. 2. With our selected AO we searched for literature that assessed endpoints relating to key events (KEs) leading to inflammation-derived lung fibrosis using a matrix-based search strategy (detailed in Additional file 1), and created a data library of models assessed with their respective doses applied and endpoint data identified. Within the AOP description, the KE “Increased, secretion of pro-inflammatory mediators” (chosen as the KE of interest for in vitro studies (Fig. 1)), identifies a number of possible mediators including cytokines (e.g. IL-1, IL-6 and TNF families), chemokines, and growth factors (e.g. TGF-β and PDGF). By using general terms in our search strategy (See details in Additional file 1) we aimed to include as many (pro-)inflammatory mediators as possible to provide a broad range of useful comparisons for IVIVE.

Two important stages in our IVIVE methodology were to 1) identify a relevant approach to align dose metrics of in vivo and in vitro systems and 2) to identify any associations between effect–responses (endpoints) resulting from particle exposures. (1) For in vivo systems the exposure was represented in relation to affected tissue surface area, in this case surface area of the proximal alveolar region, as defined by Donaldson et al*.* [22], with exposure doses being estimated either by modelling of particle lung deposition, or based on measurements of particle lung burden data; we also selected exposure per tissue surface area (i.e. the area of an exposure plate) within in vitro systems, instead of exposure per fluid volume, as this allowed comparisons to be more easily interpreted and transferable [12]. In each case, this enables focus on the affected region. (2) To align effect–responses (endpoints) resulting from particle exposures, endpoint data which sufficiently represented a dose-dependent response were assessed via statistical analysis (log–log regression models, benchmark dose (BMD) analysis, and EC50 determinations) to establish the degree of concordance between selected in vivo data and in vitro data sets.

## Data collection on particles

For the model scenario of inflammation-derived lung fibrosis, α-quartz was chosen as this particle is well-defined as following this mode of action. The studies included utilised either DQ12 (Dorentrup kaolinitic sand deposit in Westphalia, Germany) or silica (Min-U-sil^®^ 5 Ground, U.S. Silica Company, Berkeley Springs, WV, USA), as these are the most common α-quartz particles used in literature and have been well-characterised for their physico-chemical properties [23, 24]. For our case study material nano-CeO_2_ was chosen as in vivo studies have shown a risk of pulmonary fibrosis [25, 26]. Details of the literature screening method can be found in the Additional file 1.

Literature on the toxicity of α-quartz and nano-CeO_2_ relevant to the fibrosis AOP (Fig. 1) were screened to identify and extract suitable in vivo and in vitro endpoint data (e.g. PMN influx, cytokine release by lung cells, development of lung fibrosis via histopathology scoring and hydroxyproline levels [27, 28]). Information on the study protocol was also considered to allow meaningful comparisons. For this proof-of-concept, we limited the in vivo studies to rat inhalation only to obtain a more accurate representation of dosimetry in vivo allowing a more robust dose comparison (Step 1). Additionally, both in vivo and in vitro articles were excluded if the endpoints assessed had no relevance to the fibrosis AO and/or if there were insufficient information to proceed with data assessment (e.g. no details on number of replicates, missing information on the study protocol).

The in vivo studies included in the assessment were limited to rat inhalation studies, whereby the animal was exposed either by whole-body, head-only or nose-only methods. Other exposure methods, such as instillation, were not included as these have been shown to cause different distribution of particles in the lung and can result in increased inflammatory response when compared to inhalation exposure [29]. Studies were used if they reported PMN influx either in PMN numbers (e.g. 10^6^ cells/rat) or relative PMN % from total cell number, or if sufficient details were included to allow conversion to these units. Additionally, we limited studies to those who reported lung burden values (and had reported the methodology for doing so). As discussed briefly above, for α-quartz the in vivo studies available in the literature reported units of PMN influx in number of cells counted (namely 10^6^/rat), and none contained the sufficient information to allow conversion to % of total cells. This is not ideal as errors will be present due to the different methods used for counting cells. To reduce errors due to methodology differences, as well as operator differences, it would be better for these units to be converted to % of total cells.

For in vitro studies, we included all possible lung cellular models, including both submerged systems and air–liquid interface (ALI) systems. Studies were excluded if they were deemed to be not of sufficient quality, i.e. they did not contain information such as the number of biological replicates used to create averages, not reporting the units used, or missing information on the protocols used. Additionally, we limited the studies to those that reported applied dose as a mass per surface area unit (µg/cm^2^), or contained all the relevant information to allow this conversion from a mass per volume concentration (µg/ml), such as the volume applied, the surface area of the well or details of the plate used. We also assume that all particles in a suspension used for submerged exposure conditions will deposit on the cells before the marker of toxicity/response was measured.

Endpoint data was extracted directly from tables with information on averages and standard errors, or from graphs using the online program WebPlotDigitizer (v4.5) [30].

## Aligning in vivo and in vivo dosimetry

### Step 1: conversion of in vivo dosimetry to mass per surface area of tissue

To allow effective comparisons between in vivo and in vitro studies, comparable dosimetry was required. We have selected studies which allow interpretation of dose based on measured lung burden of α-quartz or nano-CeO_2_; as this followed inhalation exposure, we are also able to determine in vivo dosimetry through modelling lung deposition. To compare the two methods, for our model scenario, a mass-based dosimetry for in vivo studies was determined by both obtaining the retained lung burden (i.e. α-quartz concentration measured in lung tissue), and by modelling the lung deposition using Multiple-Path Particle Dosimetry Model (MPPD) v3.04 [31] with the parameters detailed in Table 1. Due to the irregularity in morphology of quartz particles, equivalent diameter model was applied. The average value of deposition fraction for particle MMAD 1.4–2.0 µm (range based on values defined in the in vivo studies used [27, 28, 32]) was given as 12.2% in the airways (tracheobronchial and alveolar regions) and 2.5% in the alveolar region. Therefore, estimated deposited dose was calculated using the exposure dose, deposition fraction, a rat inhalation rate of 0.25 L/min (0.015 m^3^/h) [33] and the total exposure time in hours, as detailed in the following equation:

$$Deposited\;dose \left( {mg} \right) = Dose\; \left( {mg\,m^{ - 3} } \right) \times Inhalation\;Rate \left( {m^{3} \,h^{ - 1} } \right) \times Exposure \;time \left( h \right) \times Deposition \;fraction$$

**Table 1:** Parameters used in MPPD modelling of rat lung deposition of α-quartz

MPPD model input parameters	
Species	Rat
Model	Symmetric Sprague Dawley
Body weight (g)	450
FRC volume (mL)	Default for Sprague Dawley rat (4.3578 mL)
URT (Head) volume (mL)	Default for Sprague Dawley rat (0.53668 mL)
Density (g/cm^3^)	2.6
Diameter (MMAD) (µm)	1.4, 1.6, 1.8, 2
Geometric SD (µm) [32]	2
Concentration (mg/m^3^)	1
Breathing frequency (min^−1^)	Default for Sprague Dawley rat, nose only exposure (166 per minute)
Tidal volume (mL)	Default for Sprague Dawley rat, nose only exposure (3.37267 mL)
Inspiratory fraction	Default for Sprague Dawley rat (0.5)
Pause fraction	Default for Sprague Dawley rat (0)
Breathing scenario	Nose only exposure
Clearance rate (day^−1^)	Deposition only

 In this comparison, PMN influx data related to the given lung burden was taken with no post-exposure conditioning, therefore minimal clearance would have occurred following termination of the study. However, as the deposited dose calculated is based on multi-day experiments some level of clearance will have occurred, which is highly dependent on study duration. This has not been accounted for in our calculation, therefore establishing the clearance rate of a particle in the lung throughout the study duration would be essential to allow modelling of the particle deposition, if measuring retained lung mass is not possible.

**Fig. 3:**
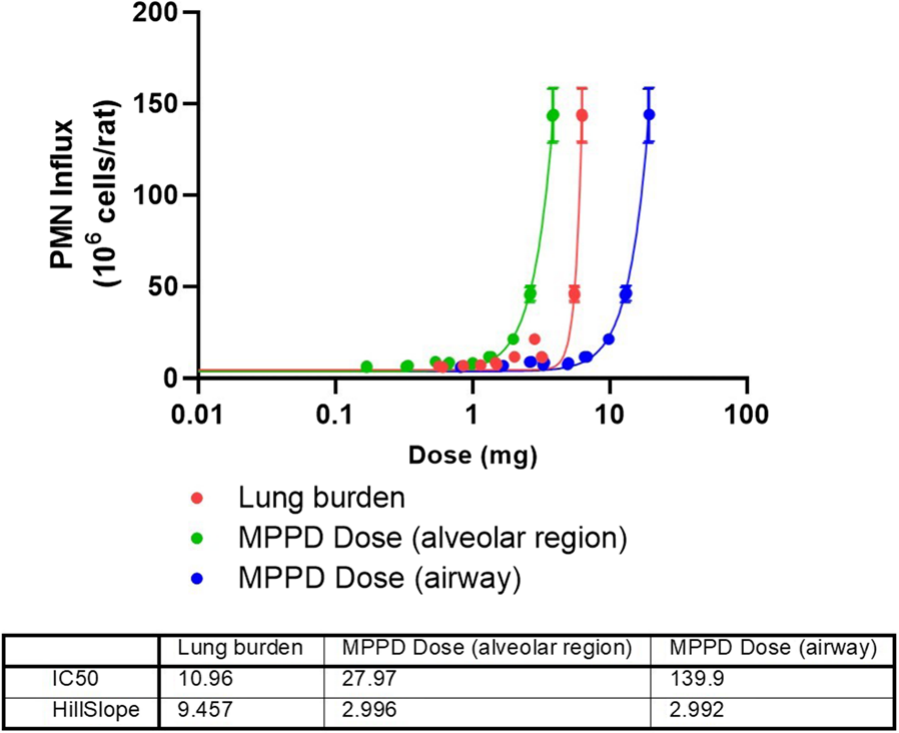
PMN influx following exposure to α-quartz. Data chosen for IVIVE is represented by the different methods of calculating extrapolated dose. Dose corresponding to retained lung burden values (red), or calculated using MPPD airway deposition (blue), and MPPD alveolar deposition (green). Best-fit IC50 and Hillslope values provided were calculated using nonlinear regression “Sigmoidal, 4PL, X is concentration” analysis in GraphPad. Data points given are mean ± SEM

Figure 3 shows the difference in the dose–response curves for PMN influx using in vivo α-quartz dosimetry determined by measuring lung burden or modelling the lung burden in either the airway or only the alveolar region by MPPD. The use of retained lung burden measured at the same time as the endpoint in question (in this case PMN influx) is the preferred option in our opinion as this reflects the true delivered dose necessary to elicit the response. In the following analysis, measured lung burden has been chosen to test our methodology. However, the use of modelling can provide a useful alternative if retained lung burden is not available. As also discussed by Ma-Hock et al*.* [12], the use of MPPD-estimations would incur additional uncertainty in the result, the magnitude of which would be determined after successful validation of the IVIVE method.

Once a mass-based dosimetry had been determined, the in vivo dose was then converted to µg/cm^2^ by dividing the mass delivered to the rat lungs by the surface area of the proximal alveolar region (300 cm^2^ [22]), to align dosing descriptions across all models. This value was chosen as it is an estimate of the surface area of the proximal alveolar region (PAR) of a rat, where particles are retained. Similarly for in vitro models, as explained above in the ‘Data collection on particles’ section, the dose was already expressed as a unit per surface area of the exposure well, and if not we converted it to such, given the right parameters reported, following recommendations of Ma-Hock et al*.* [12] for setting in vitro doses based on in vivo responses.

### Step 2: conversion of dosimetry units to reflect the surface-driven effect

As has been done by others [12, 22, 34–36], we have compared doses using a surface area (of particle)-per surface area (of proximal lung tissue) derived dose in cm^2^/cm^2^, which also allows us to take into consideration variation in the α-quartz particles assessed as well as the assumption that inflammation-derived fibrosis is a particle surface-driven effect. Therefore, where information was available to do so, all in vitro nominal doses were converted to cm^2^/cm^2^ by multiplying the mass-per surface area of exposure dish concentration (µg/cm^2^ i.e. 10^–6^ g/cm^2^) by the specific surface area (SSA) of each particle (in m^2^/g i.e. 10^4^ cm^2^/g) with a conversion factor of 0.01 (due to the unit conversion):

$$Dose \left( {cm^{2} \,cm^{ - 2} } \right) = mass \;per \;surface \;area\; dose \left( {\mu g\,cm^{ - 2} } \right) \times BET \;surface\; area \left( {m^{2} \,g^{ - 1} } \right) \times 0.01$$

Similarly, the in vivo dose, now in retained mass per proximal rat lung surface area (µg/cm^2^) as described in Step 1 above, was also multiplied by the SSA of each particle to obtain a surface area derived dose in cm^2^/cm^2^. The SSA values for DQ12 and Min-U-sil^®^ 5 were taken from literature as 10.1 and 5.1 m^2^/g, respectively [23, 24] and for nano-CeO_2_ SSA values were taken directly from the paper, or in the case of known JRC reference materials NM-211 and NM-212 from Singh et al*.* [37]. These values were obtained by nitrogen gas adsorption method (Brunauer–Emmett–Teller (BET) analysis). BET has previously been identified as providing an overestimation of biologically relevant surface area [12], in particular when pores are present in the material or voids are created within agglomerates. However, due to the heterogeneous nature of the quartz particles chosen these SSA values are used only for the purposes of aligning tests performed with DQ12 and Min-U-sil^®^ 5, therefore any errors induced by the measurement technique were not believed to be significant.

Using the dosimetry alignment outlined in Steps 1 and 2, we were then able to plot the dose–response relationship for the in vivo PMN influx using a dose easily translatable to in vitro exposures.

## Comparing endpoints

### Step 3: determining correlation between PMN influx and fibrosis in vivo

We began by looking at in vivo studies that investigated both PMN influx and fibrotic response in the respiratory system to determine under what conditions the influx of PMN was associated with fibrosis, and therefore used as a suitable endpoint for IVIVE. For α-quartz, much of the data obtained is from studies conducted 15 years or more ago. Standardisation of the measurement units of PMN influx was difficult as many studies did not include all information relevant to convert PMN influx to % of total cells counted, which is believed to be the most robust measurement unit as it considers the different cell counting methods utilised and hence reduces measurement errors [38, 39]. Therefore, for α-quartz we have instead restricted the PMN influx units to those which report as 10^6^ cells/rat, to improve harmonisation across different studies included. Additionally, various methods are available for identifying fibrosis. For our comparisons we have chosen to restrict this to the histopathological severity scoring method, as this is widely used [27, 28]; this resulted in four studies used for this comparison [27, 28, 32, 40].

**Fig. 4:**
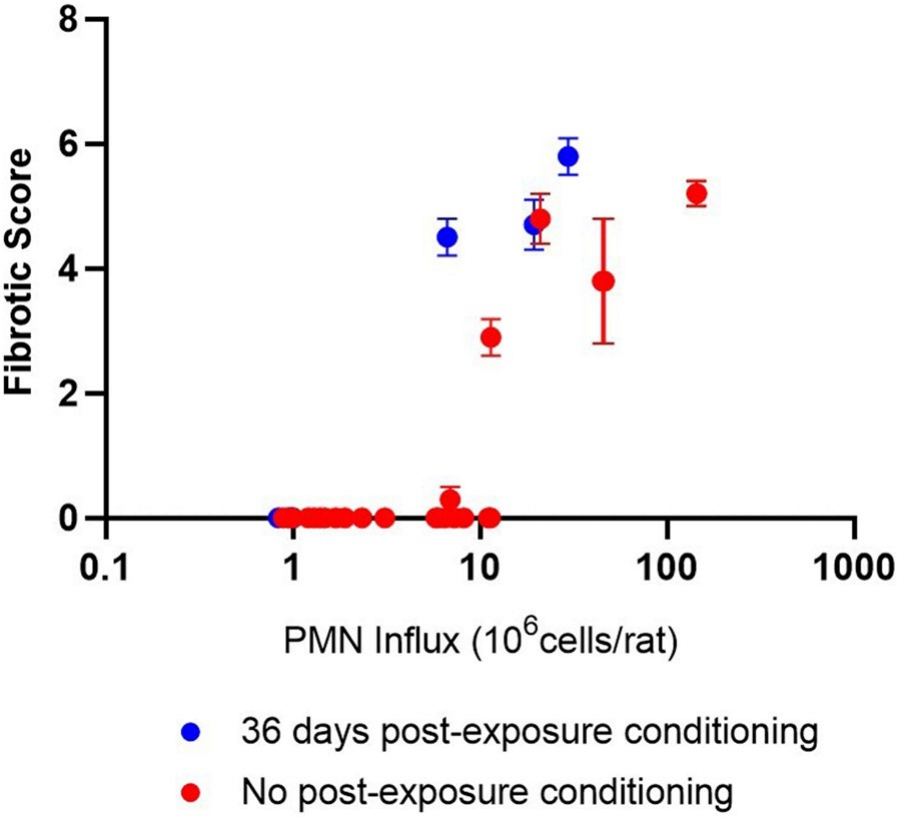
Relationship between PMN influx in vivo and lung fibrotic histopathology score following exposure to α-quartz. The α-quartz used in the source studies was Min-U-sil® 5. Blue dots are data entries where the PMN influx was recorded following a period of post-exposure conditioning (36 days), whereas red dots represent PMN influx values collected with no post-exposure conditioning time (< 48 h). Data points given are mean fibrotic score ± SEM (N = 5–6) [27, 28, 32, 40]

To ensure that the in vivo endpoint we are comparing against (i.e. PMN influx) is a sufficient biomarker for inferring the on-set of inflammation-driven lung fibrosis, the four α-quartz rat inhalation studies which reported fibrotic scores were used. Of these studies, all reported the PMN influx as 10^6^ cells/rat lung bronchoalveolar lavage fluid. The fibrotic histopathology scoring includes 8 different grades ranging from 1 (normal, no legions observed) to 8 (severe obstruction of most airways). Fibrosis is recognised at any score above 4, where minimal collagen deposition and increased bronchiolization is observed [41]. Figure 4 shows the relationship between PMN influx and fibrotic score measured in vivo. The blue dots in the graph highlight where PMN influx has been determined following a period of post-exposure conditioning, whereas red dots show only data collected with no extended post-exposure conditioning time. After the lungs reach the maximum tolerated level of pro-inflammatory conditions, a steep rise in PMN influx beyond this level is immediately followed with the progression of lung fibrosis, hence inferring that the measurement of PMN influx is a suitable marker for lung fibrosis in vivo. Focussing only on the data collected within 48 h after the exposure had finished (i.e. no post-exposure conditioning), Fig. 4 suggests that BALF PMN concentrations of > 10 × 10^6^ cells/rat have been associated with the onset of fibrosis (i.e. fibrotic score > 4) and as the PMN concentration increases above 10 × 10^6^ cells/rat the progression of fibrosis is accelerated.

### Step 4: relationship between fibrosis and study protocols

**Fig. 5:**
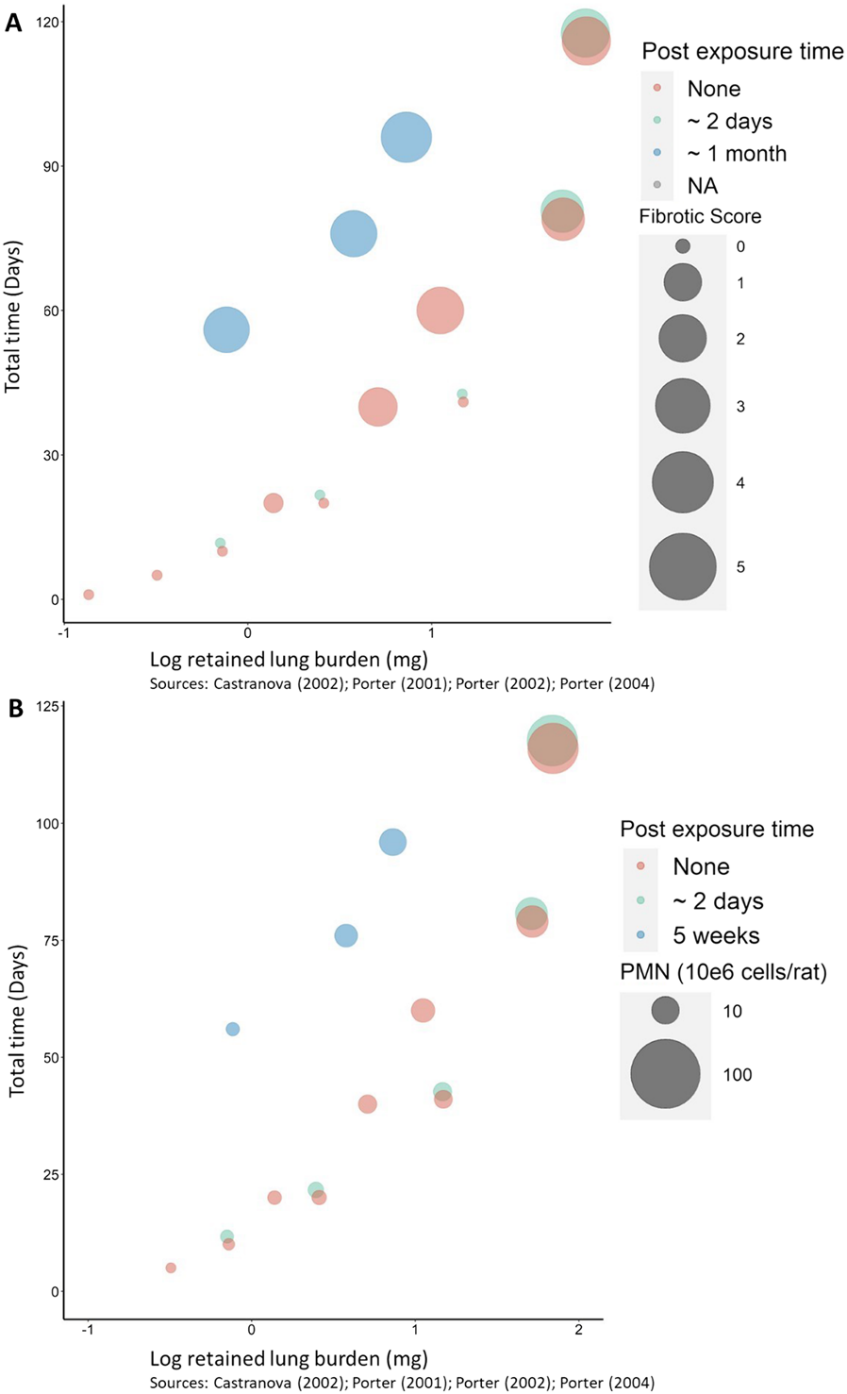
Evidence of fibrosis as a function of α-quartz exposure dose and post-exposure conditioning time. Bubble plots are separated by the method of inferring evidence of fibrosis by using A) histopathological scoring method, and B) PMN influx [27, 28, 32, 40]

To capture the effect of different dosing strategies on fibrotic response, we also mapped the fibrotic response (i.e. the severity of fibrotic histopathological scoring) as a function of both the dose applied and the exposure (and post-exposure) duration. To investigate how the relationship of both PMN influx and fibrotic score is related to the dosing schedule of the in vivo study, a bubble plot was created to indicate at which exposure doses and times an influx of PMN and fibrosis was observed (Fig. 5) [27, 28, 32, 40].

There were inconsistencies in the protocols of studies used, which has made comparisons ambiguous. To improve the interpretation of the in vivo results, we would suggest to focus on studies that utilise standardised approaches, such as OECD guidelines. The in vivo studies utilised in our approach were published between 2001 and 2004, when a limited number of standardised approaches were available. The variation in the protocols (and effects derived) within the literature assessed is outlined in Fig. 5, which shows a dose- and time-dependent relationship for increasing α-quartz (Min-U-sil^®^ 5) exposure and likelihood of lung fibrosis. As a generalisation, a pro-fibrotic effect was observed with treatments relating to retained lung burden of greater than 0.89 mg/lung, and when the analysis was performed 28 days after first exposure. Therefore, of particular interest for the onset of fibrosis, in vivo studies should be conducted following OECD Test Guideline 412 (TG 412) “Subacute Inhalation Toxicity: 28-Day Study”, and the respective PMN influx measured at various post-exposure time points (e.g. day 1 and day 28). As stated in Step 3, we suggest PMN values of > 10 × 10^6^ cells/rat are relevant for the early stages of this time-resolved fibrotic effect and represent the change in PMN influx relevant to an enhanced (acute) inflammatory response (Fig. 3).

To compare against in vitro endpoints, we have selected only the measurements of PMN concentration resulting from in vivo α-quartz exposure taken < 72 h after particle exposure had stopped. The justification for this is that our IVIVE is based on the acute inflammatory response which has the potential to develop into lung fibrosis if inflammation persists. From the data represented in Fig. 5 it is clear that as the post-exposure conditioning time increases, the fibrotic score increases far more notably than is observed for PMN influx. This suggests that time is an important factor to consider when using PMN influx as a proxy for lung fibrosis. To reduce any impact imposed by time considerations, we selected an acute inflammatory response in vivo (based on time collected after exposure, rather than total exposure time) as this better represents what is possible to test in vitro. In similar IVIVE studies [11, 12], in vivo exposure duration has also been limited to reflect a similar exposure time in vitro, however we have chosen not to include these restrictions. Instead we focus on effect (i.e. the endpoints suitable for the KE under consideration) and not time; we selected in vivo inflammation dose–response curves which are directly associated with in vivo fibrosis, and subsequently investigated associations of these in vivo inflammation dose–response curves with in vitro pro-inflammatory dose–response curves.

### Step 5: PMN influx in vivo compared with cytotoxicity and inflammatory endpoints in vitro

The comparison of PMN influx in vivo with various endpoints in vitro broadly followed two different methods. The first (Step 5a) was to compare dose response coefficients, and when aligned, to perform statistical analysis allowing dose comparisons at a central point of these curves e.g. by EC50 analysis. A second approach (Step 5b), which in theory can be applied to any curve shape, used a point-of-departure (PoD) analysis of the curves. We describe how each method could be conducted and give a discussion on when data would be suitable for each analysis type.

As discussed above, studies were excluded if the information required for robust comparisons were not included in the paper (i.e. for in vivo, only rat inhalation studies were included to allow good comparisons of the doses obtained using retained dose to MPPD modelling of dose (Step 1), and in vitro studies were excluded if information was not available to allow dose conversion to cm^2^/cm^2^. Additionally, any study would be excluded if information was not included for experimental methodology such as number of replicates or missing study protocols).

The in vitro cytokines/chemokines discussed in detail here are those that were most widely reported, and hence had the most data available (i.e., IL-8, IL-6, IL-1β TNF-α). It is recognised that other factors could be more suitable for comparison with inflammation-derived fibrosis (e.g. TGF-β), however no informative conclusions could be taken from endpoints with limited data available, hence these are not included in our discussions. Further, comparisons were based on endpoint data across the full dose range, as opposed to focussing on the steepest or maximum response range [42], given the limited amount of data.

We recognise that the following section includes some selection bias, in that we only investigate further the associations that show a dose-dependent increase in a given cytokine/chemokine. However, we feel this is justified for our test material as the aim of this section of the analysis is to select an association which agrees with the AOP we are investigating i.e. that α-quartz can induce a pro-inflammatory response at a cellular level (assessed in vitro) and that it has the potential to induce lung fibrosis in vivo (inferred by influx of PNM).

### Step 5a: comparing the magnitude of the response in vivo to in vitro for slopes determined to have similar dose-responses

To assess the degree of concordance between responses of each endpoint from particle exposure, we performed log–log regression models to compare slopes of dose–response curves (i.e. logarithmic increase in response compared to logarithmic increases in surface area standardised dose). All coefficients were exponentiated and thus represent a fold-change in the response.

Where data allowed (i.e., ≥ 4 data points), the log–log regression analysis was performed to compare the overlap of slope coefficients where there is evidence of a dose-dependent relationship for a given endpoint, and where possible, within the specific cell types and studies. To facilitate in vitro and in vivo comparisons at different dose ranges, we used (natural) log–log regression models to select those coefficients that suggested a dose–response relationship (i.e. those with confidence intervals (CIs) above 1.0). These coefficients were then compared statistically via the seemingly unrelated estimation “suest” command in Stata (v16.1), which uses a Wald chi-square test. Comparison of slopes with *P* < 0.05 was assumed to be statistically significant. This approach compares quantitatively the average magnitude (and 95% confidence intervals) of the rate of change in each model according to the specific dose ranges. This method provides additional information to the correlation analyses used previously (e.g., Rushton et al*.* [42]; Di Ianni et al*.* [43]). Moreover, the use of the steepest slope approach, as used elsewhere [11, 42, 44], was not possible for our dataset due to a lack of data points.

A summary of the results obtained from the log–log regression analysis is provided in Table 2. For the in vitro endpoint of interleukin (IL)-8 secretion, we observed a general trend of increased secretion with increasing α-quartz dose, with indicative dose–response relationships in 2/4 associations in our analysis (1.36 [95% CI 1.06–1.76] Barosova et al*.* [45]; 1.27 [95% CI 1.02–1.59] Singh et al*.* [46]). Three of seven associations represented a dose–response relationship for IL-6 secretion. For example, associations were observed for the ALI co-culture with monocyte-derived macrophages (MDMs) present (1.78 [95% CI 1.03–3.10] Barosova et al*.* [45]), submerged epithelial (3.20 [95% CI 1.44–7.10] Damby et al*.* [47]), and submerged macrophage (1.82 [95% CI 1.15–2.86] Rong et al*.* [48] particle size < 1 µm) models. One of six in vitro studies of IL-1β secretion suggested a clear response to increasing α-quartz concentrations (1.94 [95% CI 1.23–3.05], Øya et al*.* [49]) in macrophage cells. Dose–response was identified in 5/13 associations for TNF-α secretion, in submerged J774 murin macrophages (3.84 [95% CI 1.36–11.28], Boyles et al*.* [50] and 2.48 [95% CI 1.49–4.13], Nattrass et al*.* [51]), in submerged RAW 246.7 macrophage models (3.28 [95% CI 1.18–9.14], Balduzzi et al*.* [52] and [borderline] 1.16 [95% CI 1.00–1.35], Pailleux et al*.* [53]), and in submerged differentiated THP-1 human macrophage models (1.97 [95% CI 1.32–2.94], Rong et al*.* [48] particle size < 1 µm), after 24 h exposure to α-quartz.

**Table 2:** Summary of the results from log–log regression analysis for α-quartz

Criterion of comparability	Cytokine/Chemokine			
	IL-8	IL-6	IL-1β	TNF-α
Strength of dose-dependent response (overall associations)	2/4	3/7	1/6	5/13
Concordance between all cells of the same phenotypes	No overlap	No overlap	No overlap	Overlap between RAW 246.7 cells of Balduzzi et al*.* [52] and J774A.1 cells of Boyles et al*.* [50] (*P* = .393) and Nattrass et al*.* [51] (*P* = .113)
Concordance between specific cell lines	No overlap	No overlap	No overlap	No overlap
In vivo*/*in vitro* correlation*	Statistically different to in vivo response (*P* < .05)	1 study (Damby et al*.* [47]) not statistically different (*P* > .05) compared to in vivo response	Statistically different to in vivo response (*P* < .05)	Nattrass et al*.* [51] /Balduzzi et al*.* [52]: overlap with in vivo response (*P* > 0.205)

Comparison to in vivo dosimetry from retained lung burden only—not MPPD modelling dosimetry

Of these dose–response associations, only the models from IL-6 secretion from submerged A549 epithelial cells (Damby et al*.* [47]), and TNF-α secretion from submerged J774A.1 murine macrophages (Nattrass et al*.* [51]) or submerged RAW 246.7 macrophages (Balduzzi et al*.* [52]) gave overlap with in vivo PMN influx (i.e. slopes not statistically different (*P* > 0.05)).

When we try to group studies based on the cell phenotypes (e.g. all macrophage cell lines together, or all epithelial cell lines together) or specific cell lines (by comparing different studies conducted separately with a common cell line e.g. all submerged RAW 246.7 macrophage studies, or all submerged THP-1 cell studies) we note that very little concordance is observed, with overlap only noted for TNF-α secretion from macrophages, with results using RAW 246.7 macrophages from Balduzzi et al*.* [52] overlapping with that obtained using J774.A1 cells (Boyles et al*.* [50] *P* = 0.393 and Nattrass et al*.* [51] *P* = 0.113). No overlapping responses were observed between specific cell types, most likely due to limited number of studies available, whereby typically only 1 – 2 studies suggested a dose-dependent response for any one endpoint (summary findings in Table 2, full assessment detailed in Additional file 1: Table S4).

Slopes that are not statistically different to in vivo PMN influx can be analysed by making an assessment of the slopes (e.g. using EC50). The slope coefficient addresses the magnitude of the rate of change in slopes for comparison, therefore if dose–response curves follow the typical sigmoidal shape the main contributing area of the curve will be the central section where EC50 values are determined, hence should give good values for IVIVE comparisons where in vivo and in vitro slope shapes are similar.

EC50 analysis was conducted using nonlinear regression (curve fit) analysis in GraphPad Prism (version 9.3.1), using continuous, summary data with the log10(dose) in cm^2^/cm^2^ versus a normalised response. It is recognised that other models can be used to determine the half-maximal activity, such as those outlined in the ToxCast R package documentation [13], and as such when data is analysed in practice the model achieving the lowest Akaike Information Criterion (AIC) value should be used.

**Fig. 6:**
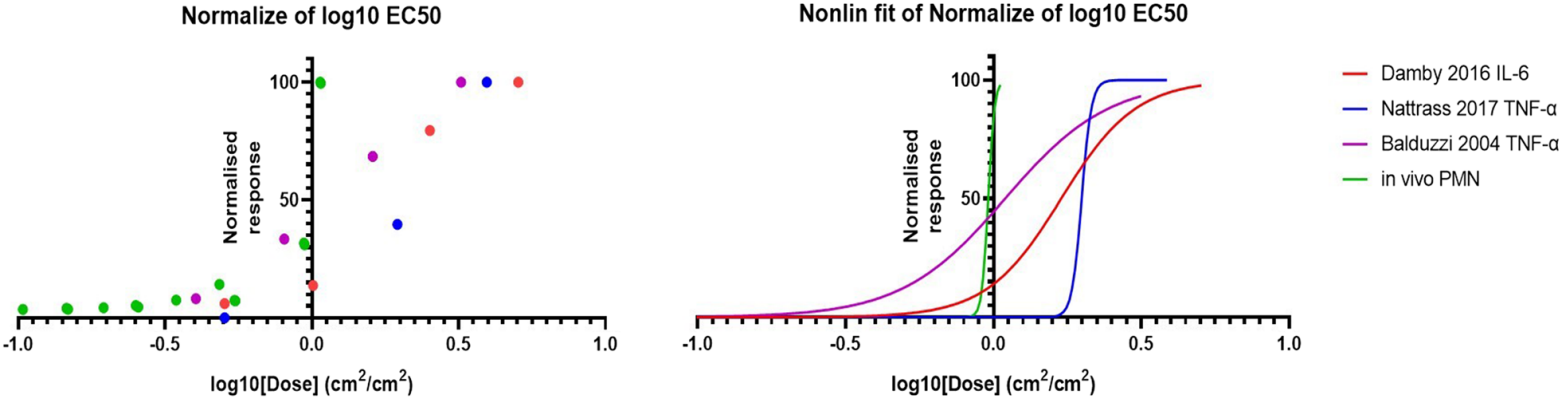
EC50 graphs for α-quartz in vivo PMN influx and in vitro inflammatory endpoints. The in vitro inflammatory endpoints included in the graphs all show overlap with in vivo PMN influx

EC50 values determined for α-quartz show good comparability between the in vivo PMN influx and in vitro cytotoxicity endpoints already identified as having good overlap in the log–log regression analysis, with all results agreeing within one order of magnitude (Table 3 and Fig. 6). However, only the response from Nattrass et al. [51] provides results with an acceptable CI range, with both Damby et al. [47] and Baduzzi et al. [52] resulting in CIs greater than one order of magnitude.

**Table 3:** EC50 results for α-quartz in vivo PMN influx and in vitro inflammatory endpoints

Dose —cm^2^/cm^2^	IL-6 in vitro	TNF-α in vitro		in vivo
log(agonist) vs. normalized response—Variable slope	Damby et al*.* [47], A549, 24 h	Balduzzi et al*.* [52], RAW 246.7, 24 h	Nattrass et al*.* [51], J774, 24 h	PAR = 300cm^2^
Best-fit value, EC50	1.695	1.095	1.988	0.9603
95% CI, EC50	0.3925 to 6.761	1.708 to ???	0.8200 to 1.452	0.9513 to 0.9683
Best-fit value, HillSlope	3.433	2.471	Unstable	40.85

The in vitro inflammatory endpoints included in the table all showed overlap with in vivo PMN influx

One of the key criteria for using EC50 analysis is ensuring that the applied dose-range results in response values within the full range between 0% effect (i.e. level of response is the same as the negative control) and 100% effect, suggested by reaching a plateau; this is particularly difficult to achieve within in vitro testing, given the tendency for higher concentrations to induce cell death and therefore reduce cytokine secretion rather than create a plateau. From the results that we have included in our study, it is not clear if this criteria has been met, particularly with regards to reaching 100% effect. This will have a huge effect on the resulting EC50 value and, in the case that 100% effect has not yet been reached, would result in an EC50 value far lower than reality.

### Step 5b: comparing point-of-departure (PoD) of the curves regardless of whether the dose–response curves have similar shapes

All dose–response curves can be assessed using PoD analysis (e.g. BMD), including those that have statistically different slope coefficients to the in vivo response. In our analysis we have chosen BMD to determine the PoD as this is a well-recognised method in human risk assessment, as also noted by Romeo et al*.* [11]. However, if other methods were used (e.g. Bayesian approach) the same considerations should be followed as described here. For BMD and statistical analysis, raw values of endpoint responses were used, where possible, as normalising data results in a loss of information on the variability and hence would increase uncertainty. For BMD analysis by PROAST, extracted dose–response data were run in the RIVM online modelling tool [54]. Continuous, summary data was utilised for all assessments, and model averaging using 200 bootstraps was applied. Where standard error of mean (SEM) or standard deviation (SD) values were not extracted (as values were too small to be seen and could not be obtained from the graph), an arbitrary small value of 1% of the average response value was assigned as SEM.

First, suitable BMR must be chosen for both in vivo and in vitro endpoints. As the in vivo data used in the IVIVE came from different studies, each following a multi-day experimental protocol, we observed high variability in the control values for PMN influx (0.7–3.1 × 10^6^ cells/rat over the course of the studies (5–116 days)). Additionally, the biological response we are wishing to observe is a high level of sustained PMN influx. Therefore we have increased the level at which a change should be acknowledged for PMN influx, compared with values used elsewhere [39, 55, 56], and have used a BMR of 100% change in response relative to background for our in vivo analysis to account for the loss in sensitivity at lower responses due to this high variability of control values and to reflect a high level of PMN influx suggestive of fibrotic potential. When we assessed PMN influx using a BMR of 100%, the results obtained provided small CIs (a small ratio of the upper (BMDU) and lower (BMDL) limits of the 90%-BMD CI is reflective of good data), however the BMDL value of 0.0464 cm^2^/cm^2^ was lower than the lowest applied dose (0.0969 cm^2^/cm^2^) (Table 4).

**Table 4:** BMD results for α-quartz in vivo PMN influx and selected in vitro endpoints

Study	Model	Endpoint	COV in controls (%)	BMR (%)	BMDL (cm^2^/cm^2^)	BMDU (cm^2^/cm^2^)	Magnitude change [log10(BMDU) – log10(BMDL)]
in vivo IVIVE	Rat	PMN	n.d.—92.62	100	0.041	0.0605	0.169
Nattrass et al*.* [51]	Submerged: J774	TNF-α	n.d	20	0.176	1.05	0.776
				50	0.449	1.37	0.484
				100	0.781	1.71	0.340
Boyles et al*.* [50]	Submerged: J774A1	TNF-α	8.33	20	0.091	0.216	0.375
				50	0.194	0.365	0.274
				100	0.344	0.531	0.189

n.d. = not determined, typically due to very low error in graphs therefore no distinction between average and error bar using software

For in vitro, the variability is likely to be dependent on both the model and the endpoint. Based on the COV (%) in the control treatments from all the qualifying in vitro models, BMRs of 20%, 50% and 100% change relative to background were chosen to identify suitability. For in vitro studies a BMR of 20% is in alignment with similar studies [11]. In general it does appear that when the COV of the control responses is lower (20% or less), the BMD analysis results in a better (less wide) BMD CI being within one order of magnitude (see in Additional file 1: Table S3).

By increasing the BMR used from 20 to 100% we see a decrease in width of the CIs (Table 4). Furthermore, for Nattrass et al*.* [51] the BMDL value now lies within the dose range applied, which is not the case at a BMR of 20%. For the current exercise it is not relevant to establish a biologically relevant BMR, since the in vitro BMD will only serve as a proxy for a biologically relevant in vivo BMD, which is derived by means of a conversion factor (see below). It should however be kept in mind that for each in vitro model-endpoint combination the same BMR is used.

Nevertheless, it does appear that responses that show a dose-dependent increase and have good quality data (i.e. low COV in controls and treatments) do show acceptable BMD CIs using a 20% BMR, as shown by the width of the CI of magnitude changes < 1 (Additional file 1: Table S3). Therefore, we suggest an additional criteria for in vitro studies that data must have COV < 20% in the negative controls to be suitable for use in calculating a conversion factor (CF) for IVIVE. The criteria of a CI range < 1 order of magnitude (i.e. tenfold difference) is arbitrary, however for subsequent use in calculating a CF a larger CI in both in vivo and in vitro BMD values would result in very large uncertainties and may result in the CF being meaningless in terms of classifying particles with respect to their likelihood of inducing lung fibrosis. When conducting in vitro tests it would be beneficial to repeat tests using a lower treatment dose-range if the current dose-range is greater than the BMD result. This would allow us to further increase the precision of the BMD result.

The distributions of the CIs in the BMD values is reflective of those observed for TiO_2_ data by Romeo et al*.* [11], in which they noted CIs extended over more than four orders of magnitude for in vitro endpoints. They also observed more precise (smaller CI intervals) for in vivo endpoints compared to in vitro, therefore we could reliably assume that the differences we observed are related to differences such as the uncertainty in the in vitro dosimetry among other experimental factors (e.g. dispersion protocols followed, time of exposures, cell concentrations). To improve the BMD results, more data points are required for both the in vivo and in vitro endpoints, and protocols followed should include quality control to ensure COV in negative controls is kept below 20%. In both in vivo and in vitro models, lower concentrations should be applied to the systems as the currently applied doses are typically greater than the 20% and 100% change modelled by PROAST for in vitro and in vivo endpoints, respectively. However, it is also important not to induce high levels of cell death. Therefore, doses applied should be limited to sub-lethal levels, whereby viability of > 50% appears to be suitable based on data collected by Nattrass et al*.* [51] and Boyles et al*.* [50].

If a conversion factor were to be assigned using this analysis, the corresponding BMD values for in vitro and in vivo responses would allow a rather simplistic prediction of an in vivo response based on in vitro data:

$$Conversion \;factor \left( {CF} \right) for \;IVIVE = \frac{{BMD100_{{{\text{in }}\;{\text{vivo}}\;{\text{ PMN}}}} }}{{BMD20_{{{\text{in }}\;{\text{vitro}}\;{\text{ model - endpoint}}}} }}$$

To take into account the uncertainty in this value, the CIs from both the in vivo and in vitro endpoints would need to be considered. Therefore, using the example of Nattrass et al*.* [51] with a BMR of 20% and the in vivo BMR of 100%, the conversion factor would be 0.233 with a magnitude change of 0.945. Hence, the uncertainty in this value would be a factor of 10. However, as we already stated that individual CIs within one order of magnitude would show good quality BMD results, this uncertainty in the CF could be anywhere up to two orders of magnitude when considering in vivo and in vitro CIs together. This will undoubtedly have an effect on the reliability of the IVIVE and any subsequent hazard classification of the material.

Following the rationale outlined above for determining the CF, we would expect that if a different substance were to induce a similar in vivo response as α-quartz (i.e. has the potential to induce lung fibrosis), the effects in vitro to this same substance would follow a similar pattern to that we have observed for α-quartz.

## Case Study

### Step 6: validation of method using nano-CeO_2_ as a case study material

The data extracted from studies investigating the toxicity of nano-CeO_2_ was handled in the same way as has been described for α-quartz (Steps 1–4). As detailed in Step 5, we have taken the most commonly investigated endpoints for inflammatory responses to in vitro lung models. For the IL-8 and TNF-α secretion endpoints, no dose response relationships were observed, hence no further assessment of comparison between different in vitro models nor in vivo PMN concentration could be conducted. However, for the IL-1β secretion endpoint we did observe a dose–response relationship in 1/3 associations included in our assessment (1.22 [95% CI 1.01–1.47], submerged co-culture of A549 and PMA-differentiated THP-1 cells, Cappellini et al*.* [57]), and for IL-6 secretion 3/5 associations showed a dose–response relationship including the same submerged co-culture model as highlighted in the IL-1β endpoint as well as two different nano-CeO_2_ particles tested by Kim et al*.* [58] in a submerged MH-S macrophage model (1.97 [95% CI 1.64 to 2.38] for nano-CeO_2_ (23) and 1.94 [95% CI 1.66 to 2.27] for nano-CeO_2_ (88)) (Table 5).

**Table 5:** Summary of the results from log–log regression analysis for nano-CeO_2_

Criterion of comparability	Cytokines/Chemokines			
	IL-8	IL-6	IL-1β	TNF- α
Strength of dose-dependent response (overall associations)	0/1	3/5	1/3	0/3
In vivo*/*in vitro correlation	N/A	Overall in vivo PMN influx overlaps with both Kim et al*.* [58] associations *P* = .922 and *P* = .704	No overlap *P* < .001	N/A

Overlap with in vivo PMN influx occurs only with the submerged MH-S macrophage model tested by Kim et al*.* [58] (both nano-CeO_2_ (23) and nano-CeO_2_ (88)) (Table 5).

As observed for the α-quartz data, nano-CeO_2_ in vivo and in vitro EC50 values do appear to be within the same order of magnitude. Interestingly, the in vivo EC50 value for nano-CeO_2_ appears to show inflammation occurred at lower concentrations for nano-CeO_2_ than for α-quartz, despite the respective fibrotic response in nano-CeO_2_ being lower than for α-quartz. This may be an artefact of data collection such as the difference in PMN influx reporting from number of PMN cells to % of total cells counted, study protocol timings or not reaching 100% response; however, it is also possible that this is a true result (i.e. nano-CeO_2_ does induce higher inflammation) but that other factors also contribute to the fibrotic potential, such as the retention time of particles in the lung. This discussion is outside the scope of this paper, however should be further investigated.

**Fig. 7:**
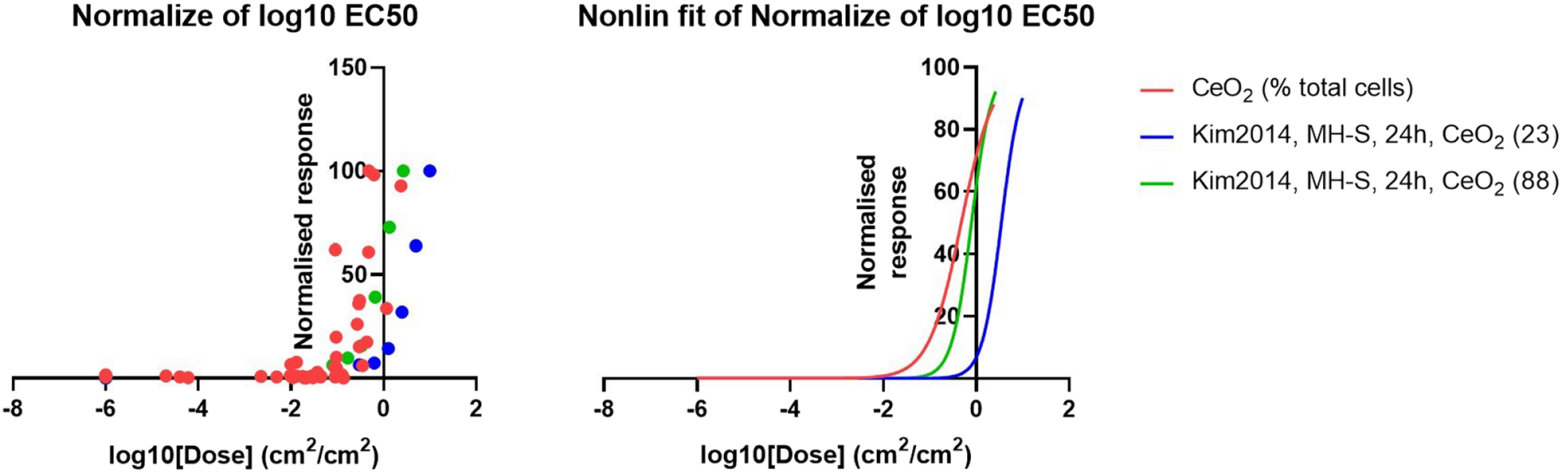
EC50 graphs for nano-CeO_2_ in vivo PMN influx and in vitro inflammatory endpoints. The in vitro inflammatory endpoints included in the graphs all show overlap with in vivo PMN influx

The EC50 results determined for in vivo PMN influx and the in vitro IL-6 endpoints by Kim et al*.* [58] were all within one order of magnitude, and have acceptable CIs (Fig. 7 and Table 6). Interestingly, the two CeO_2_ nanoparticles tested by Kim et al*.* [58] result in quite different EC50 values, despite dosimetry being converted to cm^2^/cm^2^ (which should take into account differences in surface area of particle). This would suggest a difference in activity of the two particles, related to more than just the particle size and surface area.

**Table 6:** EC50 values for nano-CeO_2_ in vivo PMN influx and in vitro inflammatory endpoints

Dose — cm^2^/cm^2^	IL-6 in vitro		in vivo
log(agonist) vs. normalized response—Variable slope	Kim et al*.* [58], MH-S, 24 h, CeO_2_ (23)	Kim et al*.* [58], MH-S, 24 h, CeO_2_ (88)	PAR = 300 cm^2^
Best-fit value, EC50	3.452	0.7873	0.4742
95% CI, EC50	3.105 to 3.830	0.7039 to 0.8788	0.3648 to 0.6544
Best-fit value, HillSlope	2.101	2.055	1.242

The in vitro inflammatory endpoints included in the table all showed overlap with in vivo PMN influx

As outlined in Step 5 for α-quartz, when an endpoint shows a dose-dependent increase in response using the log–log regression analysis and data is of good quality, BMR 20% values calculated for the in vitro studies generally showed acceptable CIs (magnitude change < 1). Therefore, the BMR 20% for in vitro endpoints that showed a dose-dependent increase using the log–log regression analysis for nano-CeO_2_ were compared with the BMR 100% values obtained for in vivo PMN influx (Table 7). Similarly to α-quartz, the nano-CeO_2_ in vitro data showed larger CIs than the in vivo combined PMN influx, however for the responses chosen, in general the CIs were within the acceptable range with two showing values only slightly greater than one order of magnitude, despite 3/4 responses having COV (%) of controls > 20%.

**Table 7:** BMD results for nano-CeO_2_ PMN influx in vivo and cytokine endpoints in vitro

Particle	Study	Model	Endpoint	COV in controls (%)	BMR (%)	BMDL (cm^2^/cm^2^)	BMDU (cm^2^/cm^2^)	Magnitude change [log10(BMDU) – log10(BMDL)]
Nano-CeO_2_	in vivo IVIVE	Rat inhalation	PMN	n.d.—99.08	100	0.117	0.287	0.390
Nano-CeO_2_	Cappellini et al*.* [57]	A549 + dTHP-1 (sub.)	IL-1β	42.23	20	0.593	7.4	1.096
Nano-CeO_2_	Cappellini et al*.* [57]	A549 + dTHP-1 (sub.)	IL-6	12.23	20	2.44	7.92	0.511
Nano-CeO_2_ (23)	Kim et al*.* [58]	MH-S	IL-6	43.30	20	0.00651	0.0833	1.107
Nano-CeO_2_ (88)	Kim et al*.* [58]	MH-S	IL-6	43.30	20	0.00335	0.0322	0.983

The in vitro endpoints included are those that show dose-dependent increases following exposure to nano-CeO2. BMDL/U20 are given for the in vitro endpoints and BMDL/U100 values are provided for in vivo PMN influx

The range of BMD values calculated for the various in vitro endpoints varies by almost 3 orders of magnitude. This highlights the differences observed for different in vitro models. The BMD CIs obtained for the Kim et al*.* [58] studies were relatively imprecise, therefore, as suggested in Step 5, more investigations would be required to determine a more precise BMD for this model, e.g. by extending the dose-range or making efforts to reduce the COV (%) of negative controls.

## Discussion

Scaling of the in vivo dosimetry to the same units as in vitro (cm^2^/cm^2^) using the approximate surface area of the proximal alveolar region (300 cm^2^ [22]) was successful, and allowed useful comparisons of the in vivo and in vitro endpoints. The comparisons of dose response curves identified promising in vitro endpoints for IVIVE, whereby studies investigating the IL-6 and IL-1β cytokine response gave particularly promising correlations with in vivo PMN influx for both α-quartz and nano-CeO_2_ data. Interestingly, for both particles it was apparent that the cytokine responses from simple submerged systems had the best correlation with in vivo PMN concentrations, with macrophage-only models most often giving best results for these poorly soluble granular particles. Although some positive results were observed for the ALI co-cultures described by Barosova et al*.* [45], the added complexity of co-culture and/or exposing using ALI model does not appear to give added benefit for the KE of interest in our study. This conclusion has been discussed by others [29] and is a particularly useful finding when considering principles such as safe-by-design, which benefit from having well-established simple methodologies available to screen possible particles before further use in processes and/or products.

One of our main research questions was whether investigating particular KEs in vitro is sufficient to infer the onset of the chosen AO. From our results it could be suggested that indeed simple in vitro macrophage models can be used to infer the onset of lung fibrosis. However, we do note that not all associations included in our study resulted in a dose-dependent increase in the secretions of cytokines investigated (IL-8, IL-6, IL-1β and TNF-α), and variability in the EC50 and BMD values calculated shows vast differences in the models used. Moreover, it is possible that the simple macrophage model is too sensitive and doesn’t accurately describe the in vivo effect. It would be prudent to investigate this further by testing the chosen model with various particles, including those that are not suspected to give an immune response in humans, to determine if false positives would be detected. Moreover, future use of these screening techniques would benefit from the inclusion of both positive and negative controls relevant to the AOP of interest to allow ranking of particles [29, 59].

We noted large variability across studies, even when utilising the same cellular models, both between the different endpoints (IL-6, IL-1β and TNF-α) and between studies investigating the same endpoint. Additioanal file 1: Tables S4 and S5 outline the comparisons made between models using the same cell phenotypes (e.g. macrophages) and additionally using the same cell line (e.g. RAW 246.7 macrophages) for α-quartz and nano-CeO_2_, respectively. For nano-CeO_2_, the limited number of studies included meant that very few valuable comparisons could be made. For α-quartz we found that the TNF-α secretion endpoint was the only in vitro endpoint showing overlap of dose–response associations across cell types (macrophages); however, no overlap within cell types was identified for any endpoint. This may be due to procedural differences, such as variation in the exposure duration, differences in dispersion protocols, different instrument sensitivity or simply differences in handling of the cells. As outlined in the relevant analysis sections, we also found large variability due to the lack of data points in each individual study (typically four or fewer), therefore extending the dosing range would greatly enhance our statistical analysis.

Our assessment was also limited to what was available in the relevant literature. This meant that we were unable to make appropriate assessments with endpoints that may be more sensitive for this KE, including that of profibrotic mediators TGF-β and PDGF. In the paper by Halappanavar et al*.* [9], it is recommended that any testing strategy for assessing relevance to an AOP should include the assessment of multiple endpoints, whereby each individual endpoint may have different sensitivities. This could be accounted for in our assessment by assigning each endpoint a relative “sensitivity factor”, which would facilitate the derivation of a single PoD or EC50 value, or at least an appropriate range. For example, if IL-1β was defined as the most sensitive endpoint we could assign this a sensitivity factor of 1 (i.e. maximum expected response), then if it is also consistently shown that IL-6 is four times less sensitive than IL-1β (as is the case with the PoD data for nano-CeO_2_ from Cappellini et al*.* [57]) we would assign IL-6 a sensitivity factor of 0.25. If we apply this to our PoD data for nano-CeO_2_ from Cappellini et al*.* [57] then we achieve BMDL values of 0.6 cm^2^/cm^2^ for both IL-1β and IL-6 (accurate to 1 significant figure only). However to do this well, responses from various different particles would need to be assessed, all following the same protocol. Since this information is not currently available, we were unable to assign any “sensitivity factors” to the endpoints we determined to be promising (IL-6 and IL-1β secretion from submerged macrophages).

As seen in our results, derivation of an extrapolation value from in vitro endpoints would result in a range of estimated in vivo responses which would span across at least one order of magnitude, with the associated CIs resulting in very high uncertainties. This level of sensitivity would not be acceptable in chemical risk assessment as ultimately we would not be able to classify the hazard of a material with any confidence. To improve the robustness of the in vitro assays, assessments should follow standardised protocols such as those developed in recent projects such as PATROLS and NanoValid [4, 60], which have shown good correlation between different laboratories during round-robin testing. Also, using suitable dosing concentrations and exposure durations would improve variability across studies, as some associations may only show no response due to the concentrations chosen in testing. However, consideration should also be given to possible interference of particles with assay read-outs [12], by including appropriate inference tests.

The results obtained from our analysis would most certainly improve if all studies included worked with harmonised protocols, reducing errors caused by such methodical differences as the dispersion protocol, exposure time and reporting units. For example, it was noted that for in vitro studies many different cell culture medium (CCM) were used, some of which included the use of proteins (e.g. BSA and FCS) to prepare stable particle dispersions for submerged cultures. The presence of proteins in CCM will result in the formation of protein corona, which is likely to alter the responses we are investigating. Therefore it would be prudent to investigate further the impact this has on the endpoints of interest to us, and ultimately to determine which experimental conditions provide the closest similarity to in vivo PMN influx. The in vivo data utilised in our assessment of α-quartz were from historical studies (> 15 years). It would be preferential to repeat these experiments using more modern methodology and practices, in particular making sure that the studies followed standardised methodology (e.g. OECD Test Guideline 412) and that the data reported is FAIR (Findable, Accessible, Interoperable and Re-usable) [61].

It is also worth noting that the exposure dose for submerged models in vitro was assessed only as the dose administered to the exposure dish, and no modelling nor experimental assessment was made of the dose delivered to the cells. This is due to a lack of information provided in the studies used within this assessment, whereby none of the in vitro studies included any modelling of delivered dose (e.g. by methods outlined by Deloid et al*.* [62] and Botte et al*.* [63]). Additionally, α-quartz powder is known to have a highly heterogeneous particle size distribution, therefore it is likely that any modelling conducted would give varying results. However, it is generally accepted that use of deposited dose in vitro gives better correlation to in vivo results [11], and in our model would give increased relevance when comparing to the lung burden values as both would be considered to be the true internal dose. The advantage of using air–liquid interface (ALI) cell exposure systems is that they typically utilise quartz microbalances, and hence give a far better representation of the delivered dose. With this in mind, successful inter-laboratory comparisons should include two phases; (1) determine if the deposited dose can be reproduced, and (2) see if the cellular response can be reproduced. To make these IVIVE comparisons robust, future work must focus on successfully achieving reproducible results in both phases, rather than focussing only on the reproducibility of cellular responses.

Analysis of the case study material (nano-CeO_2_) compared with α-quartz showed some potential for the approach we have utilised as we were able to find in vitro endpoints that could be compared statistically to the respective in vivo PMN influx endpoint. For the EC50 calculations we noted nano-CeO_2_ resulted in a lower EC50 value than α-quartz using the in vivo PMN influx. This may be due to the errors that can arise in calculating EC50 values, namely the data reported not fitting the full sigmoidal dose–response curve from 0 to 100% response effect. However, this may also reflect some additional considerations for the approach, such as including the impact of physico-chemical effects on the probability of the AO (e.g. particle persistence). For both particles, the BMD analysis highlighted that an area requiring further investigation is the determination of a biologically relevant response for the various in vitro endpoints, which may be facilitated by assessing the response to models from particles known to not cause any inflammatory response.

The IVIVE approach discussed in this paper works on the ideal scenario whereby we already know that lung fibrosis occurs in humans and rats upon exposure to α-quartz, therefore inter-species extrapolation is justified. This means that for the lung fibrosis AO, we were able to use a regulatory accepted method for assessing particle toxicity (i.e. inhalation in vivo) as a proxy for human particle toxicity in our comparisons against in vitro endpoints. This will not always be the case for different AOPs [64]; therefore, consideration must be taken for the mode of action for each chemical and to aim to assess correlation between the in vitro assays with in vivo responses in both rats and humans where possible. The AOP we have focussed on (AOP 173 for lung fibrosis) is also closely related to other AOPs [9], therefore if only looking at one KE as we have, it must be considered that a positive correlation with the chosen KE may actually result in another AO such as acute inhalation toxicity, lung cancer or lung emphysema. Moreover, although our results indicate a positive correlation of cytokine secretion in macrophages in vitro to the influx of PMN in vivo, Halappanavar et al*.* [3] rightly suggest that a suitable model for assessing lung fibrosis should include various cell types to assess the full sequence of the inflammatory response. Hence, this methodology should be expanded to include different cell types and different KEs to facilitate production of a suitable in vitro strategy for determining risk of inflammation-derived lung fibrosis. Additionally, this approach would benefit from incorporating considerations on the particle physico-chemical characteristics, such as shape, aspect ratio and chemical composition, to allow appropriate conclusions to be drawn on the likelihood of sustained inflammatory response and hence the likelihood of chronic inflammation leading to fibrosis. We know that α-quartz is a persistent, highly reactive particle and hence presence in the lung tissue leads to a sustained and chronic inflammatory response, ultimately leading to lung fibrosis. For other particles, information such as particle dissolution would be relevant to include in this model to allow inference of chronic inflammation.

## Conclusions

This study successfully used the proximal alveolar surface area to scale in vivo dosimetry to a unit applicable to in vitro culture plates (cm^2^/cm^2^). This allowed us to make an assessment of whether investigating particular KEs in vitro (inflammatory cytokine secretion) is sufficient to infer the onset of the chosen AO (lung fibrosis). Although the results obtained cannot provide us with a definitive answer for this, we have identified promising associations, in particular IL-6 and IL-1β cytokine secretion from simple submerged macrophage models should be investigated further for their potential as screening tools for lung fibrosis.

We highlighted some common issues with conducting IVIVE, such as limited data availability, variability in study protocols, realistic dosimetry values (i.e. measured or modelled doses applied to cells or animals) and too few applied doses for meaningful statistical analysis (Table 8). These issues can be overcome if standardised protocols are followed, such as those already available by the OECD or those developed and validated in recent research projects.

**Table 8:** Limitations and recommendations for in vitro studies to perform informative IVIVE

**Limited data availability**
Availability of raw data files (currently scarcely available in literature)
Better recording of methodological information required for modelling. Details typically missing include number of biological replicates used to create averages, reporting units used, and missing information on the protocols such as volume of particle dispersions applied (if reported in µg/mL), the surface area of the well or details of the plate used, and dispersion method followed
**Variability in study protocols**
Harmonizing endpoint analysis with in vivo studies (i.e. same cytokines/chemokines)
Harmonizing particle dispersion protocols and aligning dose
Harmonizing cell culture and endpoint analysis protocols
**Realistic dosimetry values**
Providing information about particle deposition on lung cell surface (Dosimetry, Modelling or ALI with quartz microbalance)
**Too few applied doses**
Increase dose range for assessing dose–response relationship. This is a particular issue for ALI exposures as the process is usually very time-consuming and aerosolization limits the deposition concentration
Known hazardous materials typically only used as positive benchmarks in in vitro assays, therefore typically limited dose ranges applied

Overall, the method of extrapolating in vitro endpoints to human lung effects does look promising. Our method of aligning in vitro toxicity to the currently accepted regulatory method of in vivo rat inhalation methodology showed some good similarities. Limited epidemiological information is available for inhalation exposure to nanomaterials; however, the use of a material with known human toxicity, such as α-quartz, as a benchmark material for in vitro testing may allow suitable inference of lung inflammation. Further work in this field should focus on creating a suite of tests that are sufficient to classify lung toxicity according to various different AOPs (i.e. differentiating between lung fibrosis and lung emphysema) and to determine the severity of the effect (i.e. acute vs. chronic inflammation).

## Supplementary Information

**Additional file1.** Literature screening methodology, graphical comparisons of in vivo and in vitro responses, and data analysis results. **Figure S1.** IL-8, IL-6, IL-1β and TNF-α cytokine responses in vitro compared to PMN influx in vivo following exposure to α-quartz. **Figure S2.** IL-8, IL-6, IL-1β and TNF-α cytokine responses in vitro compared to PMN influx in vivo following exposure to nano-CeO_2_. **Table S1.** Data analysis results for in vitro studies. **Table S2.** Data analysis results for in vivo studies. **Table S3.** CIs for various BMR applied in BMD analysis. Table S4 Model comparisons for α-quartz using log-log regression analysis. **Table S5.** Model comparisons for nano-CeO_2_ using log-log regression analysis.

## Data Availability

Not applicable.
