# Supplementary material for: Establishing relationships between particle-induced in vitro and in vivo inflammation endpoints to better extrapolate between in vitro markers and in vivo fibrosis
Source: Part Fibre Toxicol. 2023 Feb 9;20:5. doi: 10.1186/s12989-023-00516-y (PMC9909881; doi:10.1186/s12989-023-00516-y)
Supplement: Supplementary file 1 — Additional file1. Literature screening methodology, graphical comparisons of in vivo and in vitro responses, and data analysis results. Figure S1. IL-8, IL-6, IL-1β and TNF-α cytokine responses in vitro compared to PMN influx in vivo following exposure to α-quartz. Figure S2. IL-8, IL-6, IL-1β and TNF-α cytokine responses in vitro compared to PMN influx in vivo following exposure to nano-CeO2. Table S1. Data analysis results for in vitro studies. Table S2. Data analysis results for in vivo studies. Table S3. CIs for various BMR applied in BMD analysis. Table S4 Model comparisons for α-quartz using log-log regression analysis. Table S5. Model comparisons for nano-CeO2 using log-log regression analysis. [file 12989_2023_516_MOESM1_ESM.pdf]

Supplementary Information

Literature screening

A strategic literature search has been conducted to obtain the most relevant information for inflammation-derived lung fibrosis. Key search terms and phrases were formulated based on methods and the biological and chemical processes which represent key stages in AOP 173 for inflammation derived lung fibrosis, and used alongside search terms specific to materials of interest. A matrix-based search strategy was developed, using Boolean logic operators (AND, OR and NOT), to combine the key search terms into defined search strings.

The search used the following key terms and phrases:

Specific search terms

1. ("CeO2" OR "cerium")
2. ("DQ12" OR "Min-U-Sil" OR "quartz" OR "crystalline silica")

	Specific search term 1 or 2 AND …
General Search (A)	"lung"
General Search (B)	("hazard" OR "toxic" OR "cell death" OR "cytotoxicity" OR "inflamm" OR "genotoxic") AND ("human" OR "mammal")))
General Search (C)	("hazard" OR "toxic" OR "cell death" OR "cytotoxicity" OR "inflamm" OR "genotoxic") AND ("human" OR "mammal") AND "lung")
General Search (D)	("hazard" OR "toxic" OR "inflamm") AND ("human" OR "mammal") AND "lung")

In order to gain a comprehensive summary of the available evidence, the search strategy was applied in the following databases:

- The United States National Library of Medicine: PubMed
- Thomson Reuters (formerly ISI) Web of Knowledge
- Science Direct

The references obtained from each individual search were then collated in reference manager software (Endnote). An inherent consequence of performing multiple searches across several databases is the inclusion of duplicate references in the collated dataset. Endnote was therefore used to remove duplicates in the collated set of references to form a final "computational dataset". From this list, manuscript abstracts were checked for the suitability of content and a final pool of peer-reviewed literature was downloaded (1st screening exercise). The 2nd screening exercise involved taking forward only those papers that met the criteria outlined in the "Particle data collection" section in our report.

Due to the low numbers of suitable studies, we chose to include studies referenced in various review articles included in the list after the 1st screening exercise.

Graphical comparisons of *in vivo* and *in vitro* responses

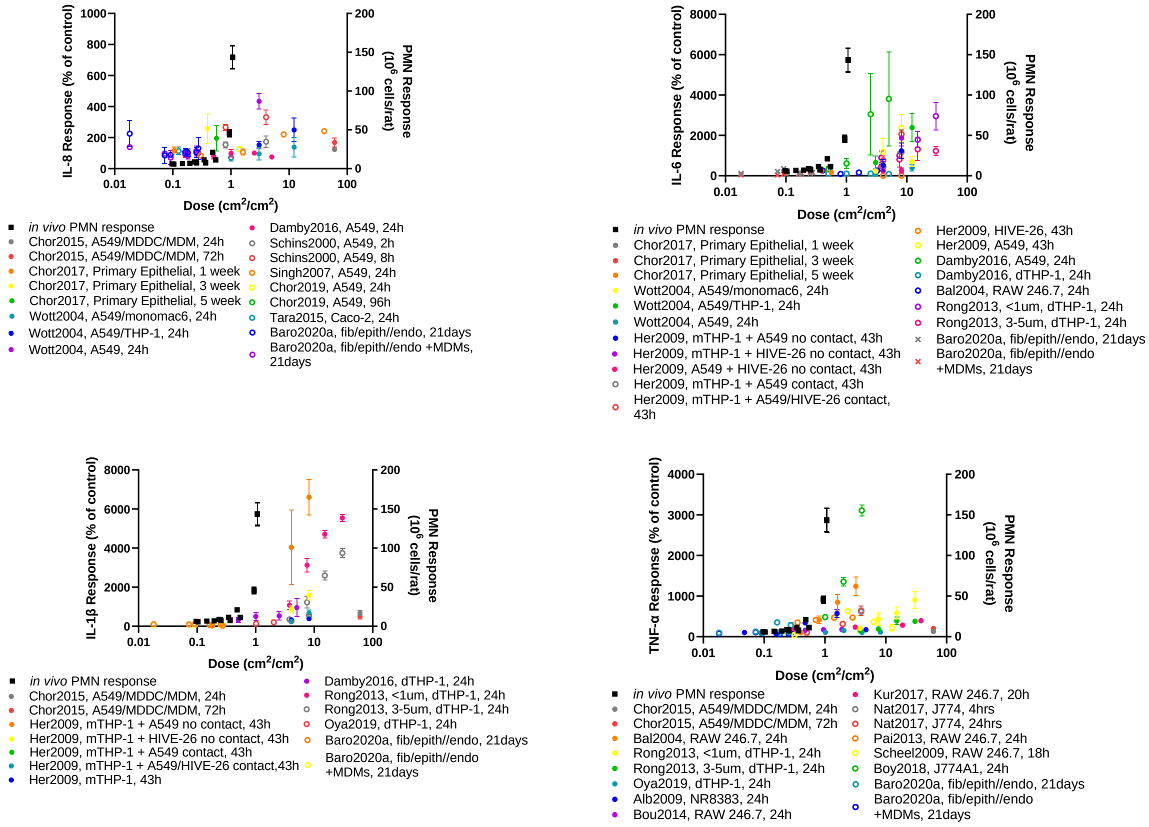

Figure S1 IL-8, IL-6, IL-1β and TNF-α cytokine responses *in vitro* [1-20] compared to PMN influx *in vivo* [21-23] following exposure to α-quartz

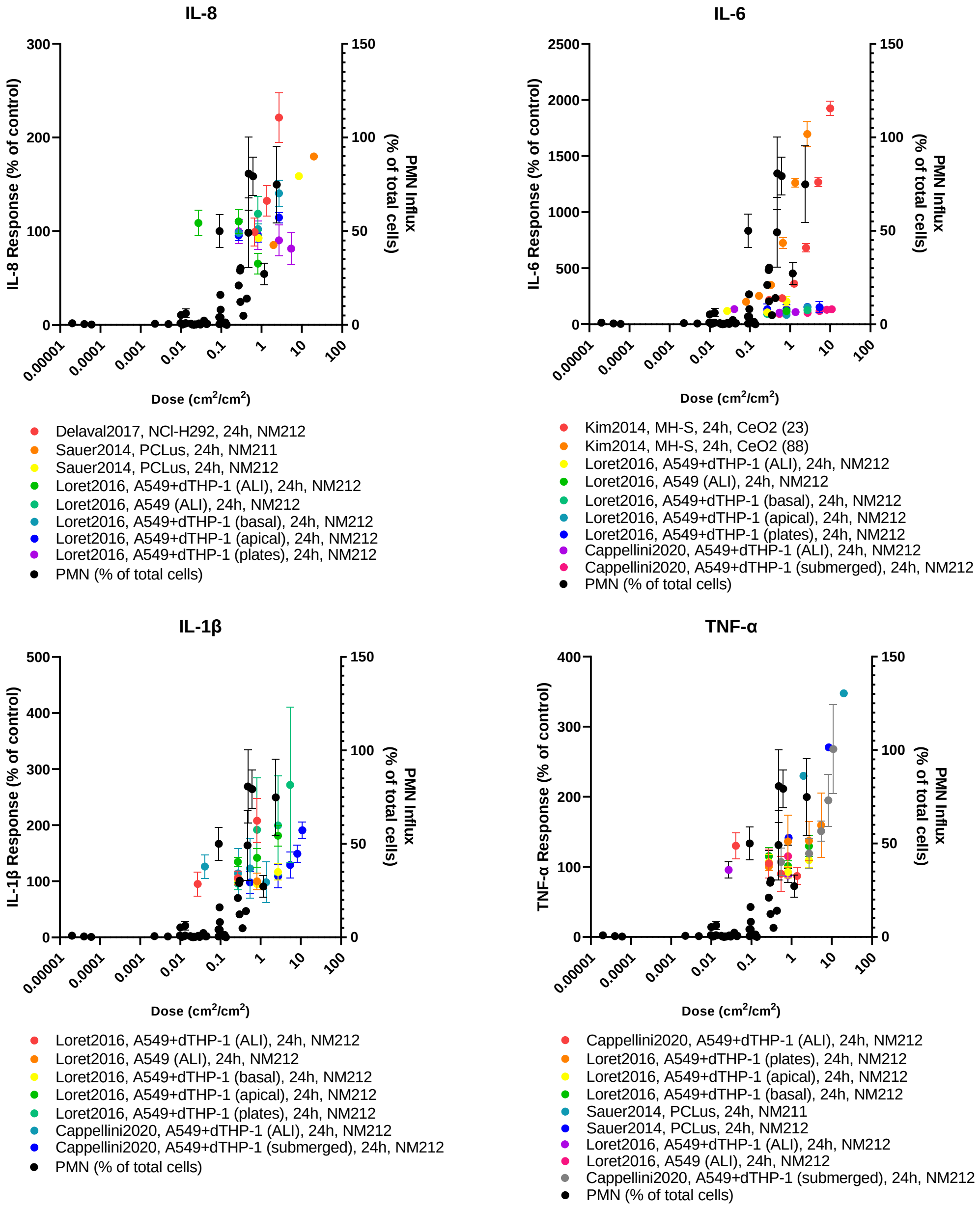

Figure S2 IL-8, IL-6, IL-1β and TNF-α cytokine responses *in vitro* [24-28] compared to PMN influx *in vivo* [29, 30] following exposure to nano-CeO_2.

Data analysis results

Table S1 Data analysis results for *in vitro* studies [1-20, 24-28, 31]

Ref	Model	Particle	Endpoint	BMR20		BMR50		BMR100		EC50	Exponentiated slope coefficient (95% CI)
				BMDL	BMDU	BMDL	BMDU	BMDL	BMDU		
Barosova et al. (2020a)	ALI: fibroblast/epithelial//endothelial	DQ12	IL-8	*	*	*	*	*	*		0.90 (0.45 to 1.78)
			IL-6	*	*	*	*	*	*		1.09 (0.73 to 1.63)
			IL-1β	*	*	*	*	*	*		0.81 (0.38 to 1.74)
			TNF-α	*	*	*	*	*	*		1.19 (0.46 to 3.06)
			TGF- β	*	*	*	*	*	*		1.34 (0.62 to 2.91)
	ALI: fibroblast/epithelial//endothelial + MDMs		IL-8	*	*	*	*	*	*		1.36 (1.06 to 1.76)
			IL-6	*	*	*	*	*	*		1.78 (1.03 to 3.10)
			IL-1β	*	*	*	*	*	*		1.14 (0.80 to 1.62)
			TNF-α	*	*	*	*	*	*		1.05 (0.65 to 1.70)
			TGF- β	*	*	*	*	*	*		1.52 (0.58 to 3.98)
Barosova et al. (2020b)	ALI: A549/THP-1//MRC-5	DQ12	IL-8	Too few data points to run any analysis							
			TNF-α								
			TGF- β								
		Min-U-Sil	IL-8	Too few data points to run any analysis							
			TNF-α								
			TGF- β								
Chortarea et al. (2015)	ALI: A549/MDDC/MDM	DQ12	IL-8	Too few data points to run any analysis							
			IL-1β								
			TNF-α								
Chortarea et al. (2017)	ALI: Primary Human Bronchial Epithelial cells	DQ12	IL-8	3.54E-06¥	0.362	0.00017¥	0.598§	0.0155¤	4.08§		
			IL-6	*	*	*	*	*	*		
			TGF- β	0.376	4.31§	0.722§	423 [Φ]	0.892§	10300[Φ]		1.06 (0.97 to 1.17)
Wottrich et al.	Submerged: A549	DQ12	IL-8	Too few data points to run any analysis							
			IL-6								
	Submerged: A549/Monomac6		IL-8								
			IL-6								
	Submerged: A549/dTHP-1		IL-8								
			IL-6								
Herseth et al.	Submerged: mTHP-1//A549	Min-U-Sil	IL-6	Too few data points to run any analysis							
			IL-1β								
	Submerged: mTHP-1//HIVE-26		IL-6								
			IL-1β								
	Submerged: A549//HIVE-26		IL-6								
	Submerged: mTHP-1/A549		IL-6								
			IL-1β								
	Submerged: mTHP-1 + A549/HIVE-26		IL-6								
			IL-1β								

	Submerged: HIVE-26		IL-6								
	Submerged: mTHP-1		IL-1β								
	Submerged: A549		IL-6								
Damby et al.	Submerged: dTHP-1	DQ12	IL-6	*	*	*	*	*	*		0.96 (0.87 to 1.07)
			IL-1β	1.80E-06¥	0.18¤	9.69E-06¥	0.408¤	8.03E-04¥	1.01		1.51 (0.97 to 2.38)
	Submerged: A549		IL-8	*	*	*	*	*	*		0.98 (0.64 to 1.50)
			IL-6	1.89E-04¥	0.194¤	0.00221¥	0.332¤	0.0123¥	0.603	1.695	3.20 (1.44 to 7.10)
Schins et al.	Submerged: A549, 8 hours	DQ12	IL-8	Too few data points to run any analysis							
	Submerged: A549, 2 hours		IL-8	Too few data points to run any analysis							
Singh et al.	Submerged: A549	DQ12	IL-8	1.03	4.23	1.9	5.91	3.25	8.77		1.27 (1.02 to 1.59)
Chortarea et al. (2019)	Submerged: A549	Min-U-Sil	IL-8	Too few data points to run any analysis							
			TGF- β								
Tarantini et al.	Submerged: Caco-2	DQ12	IL-8	Too few data points to run any analysis							
Balduzzi et al.	Submerged: RAW 246.7	Min-U-Sil	IL-6	*	*	*	*	*	*		1.09 (0.20 to 6.06)
			TNF- α	0.00144¥	0.179¤	0.00533¥	0.328¤	0.0149¥	0.454¤	1.095	3.28 (1.18 to 9.14)
Rong et al.	Submerged: dTHP-1	DQ12 <1 µm	IL-6	2.00E-06¥	0.108¥	1.93E-05¥	0.385¤	4.32E-04¥	0.862¤		1.82 (1.15 to 2.86)
			IL-1β	0.183¥	0.664¤	0.433¤	1.08¤	0.738¤	1.51¤		2.16 (0.88 to 5.31)
			TNF- α	0.00146¥	1.22¤	0.0248	2.63¤	0.181	5.06		1.97 (1.32 to 2.94)
		DQ12 3-5 µm	IL-6	2.23E-06¥	0.604¤	4.78E-05¥	1.42¤	9.00E-04¥	2.28¤		1.64 (0.86 to 3.11)
			IL-1β	0.561¤	1.53¤	1.07¤	2.37¤	1.74¤	3.21¤		0.04 (<0.00 to 15.0)
			TNF- α	0.101¥	4.59	0.817¤	7.25	3.04¤	12.6		1.68 (0.94 to 3.02)
Øya et al.	Submerged: dTHP-1	Min-U-Sil	IL-1β	3.72E-02¥	1.37	0.237¤	2.41	0.754¤	3.64		1.94 (1.23 to 3.05)
			TNF- α	*	*	*	*	*	*		0.96 (0.55 to 1.69)
Albrecht et al.	Submerged: NR8383	DQ12	TNF- α	0.001¥	0.246	0.00166¥	0.37	0.0845	0.772		1.31 (0.65 to 2.65)
Boudard et al.	Submerged: RAW 246.7	DQ12	TNF- α	0.00856¥	0.19¤	0.152¤	0.403¤	8.24§	8540[Φ]		1.05 (0.94 to 1.17)
Kurtz-Chalot et al.	Submerged: RAW 246.7	DQ12	TNF- α	5.47E-05¥	0.0189¥	0.00832¥	0.22¤	0.351	2.53		1.15 (0.99 to 1.33)
Nattrass et al.	Submerged: J774	DQ12	TNF- α	0.176¤	1.05	0.449¤	1.37	0.781	1.71	1.988	2.48 (1.49 to 4.13)
Pailleux et al.	Submerged: RAW 246.7	DQ12	TNF- α	1.07E-06¥	0.0335¥	1.49E-05¥	0.082¤	7.90E-04¥	0.134¤		1.16 (1.00 to 1.35)
Scheel et al.	Submerged: RAW 246.7	DQ12	TNF- α	0.00783¥	0.637	0.0527¤	1.04	0.131¤	1.59		1.69 (0.32 to 8.85)
Boyles et al.	Submerged: J774	DQ12	TNF- α	0.091¥	0.216¤	0.194¤	0.365¤	0.344¤	0.531¤		3.84 (1.36 to 11.28)
Cappellini et al.	ALI: A549/dTHP-1	CeO_2 NM212	IL-6								0.93 (0.81 to 1.08)
			IL-1β								0.94 (0.81 to 1.09)
			TNF- α								0.89 (0.82 to 0.96)
	Submerged: A549/dTHP-1		IL-6	2.44	7.92						1.14 (1.06 to 1.22)
			IL-1β	0.593	7.4						1.22 (1.01 to 1.47)
			TNF- α								1.31 (0.99 to 1.74)

**Loret et al.**	ALI: A549/dTHP-1	CeO_2 NM212	IL-8			0.88 (0.20 to 3.90)
			IL-6			1.14 (0.16 to 8.11)
			IL-1β			1.22 (0.21 to 7.09)
			TNF- α			0.99 (0.35 to 2.74)
	ALI: A549			Too few data points to run any analysis		
	Submerged: A549/dTHP-1 (inserts basal compartment)		IL-8			1.17 (0.51 to 2.68)
			IL-6			1.13 (0.76 to 1.70)
			IL-1β			1.06 (0.45 to 2.51)
			TNF- α			1.05 (0.32 to 3.48)
	Submerged: A549/dTHP-1 (inserts apical compartment)		IL-8			1.08 (0.62 to 1.89)
			IL-6			1.24 (0.14 to 11.14)
			IL-1β			1.14 (0.64 to 2.02)
			TNF- α			1.01 (0.38 to 2.73)
	Submerged: A549/dTHP-1 (plates)		IL-8			0.94 (0.88 to 1.00)
			IL-6			1.05 (0.97 to 1.14)
			IL-1β			1.36 (0.94 to 1.98)
			TNF- α			1.14 (0.97 to 1.36)
**Sauer et al.**	PCLus test system	CeO_2 NM211<br>CeO_2 NM212		Too few data points to run any analysis		
**Delaval et al.**	Submerged: NCl-H292	CeO_2 NM212	IL-8			1.78 (0.55 to 5.78)
**Kim et al.**	Submerged: MH-S	CeO_2 (23)	IL-6	0.00651¥	0.0833¤	1.97 (1.64 to 2.38)
		CeO_2 (88)	IL-6	0.00335¥	0.0322¤	1.94 (1.66 to 2.27)

* No Model Averaging applied, due to nonsignificant trend in the ndata, ¤ Lower than applied dose, ¥ Greater than 10x lower than applied dose, § Higher than applied dose, $^{\Phi}$ Greater than 10x higher than applied dose

Table S2 Data analysis results for *in vivo* studies [21-23, 29, 30]

Ref	Model	Particle	Dosimetry	BMR100		EC50	Exponentiated slope coefficient (95% CI)
				BMDL	BMDU		
Castranova et al. 2002	Rat inhalation, 5-116 days exposure	Min-U-Sil	Measured lung burden			0.9446	3.09 (1.54 to 6.21)
Porter et al. 2002	Rat inhalation, 5-116 days exposure	Min-U-Sil	Measured lung burden			0.9496	3.26 (1.59 to 6.67)
Porter et al. 2004	Rat inhalation, 20-60 days exposure	Min-U-Sil	Measured lung burden			0.2818	3.27 (0.15 to 69.4)
Combined α-quartz	Rat inhalation	Min-U-Sil	Measured lung burden (cm^2/cm^2)	0.041¤	0.0605¤	0.9603	3.18 (2.22 to 4.54)
Keller et al.	Rat inhalation, 5 days exposure	CeO_2 NM212	Measured lung burden			0.06795	1.99 (1.43 to 2.76)
	Rat inhalation, 4 weeks exposure	CeO_2 NM212	Measured lung burden			0.3734	2.38 (0.09 to 60.1)
Schwotzer et al.	Rat inhalation	CeO_2 NM212	Measured lung burden			0.2221	1.25 (0.98 to 1.60)
Internal results (not published)	Rat inhalation	CeO_2 NM212	Measured lung burden			0.2109	2.84 (1.45 to 5.55)
Combined CeO_2	Rat inhalation	CeO_2	Measured lung burden	0.117	0.287	0.4742	

* No Model Averaging applied, due to nonsignificant trend in the ndata, ¤ Lower than applied dose, ¥ Greater than 10x lower than applied dose, § Higher than applied dose, $^{\Phi}$ Greater than 10x higher than applied dose

Table S3 CIs for various BMR applied in BMD analysis [3, 7, 9, 11-24, 28-30].

Study	Model	Particle	Endpoint	COV in controls (%)	Magnitude Change [log10(BMDU) – log10(BMDL)]		
					BMR20	BMR50	BMR100
Albrecht et al.	Submerged: NR8383	DQ12	TNF- α	22.08	2.390935	2.348094	0.960761
Balduzzi et al.	Submerged: RAW 246.7	Min-U-Sil	TNF- α	n.d.	2.094491	1.789147	1.48387
Boudard et al.	Submerged: RAW 246.7	DQ12	TNF- α	4.54	1.34628	0.423461	3.015531
Boyles et al.	Submerged: J774	DQ12	TNF- α	8.33	0.375412	0.274491	0.188536
Chortarea et al. (2017)	ALI: Primary Human Bronchial Epithelial cells	DQ12	TGF- β	7.19 – 10.53	1.059289	2.767803	4.062472
			IL-8	29.51 – 131.80	5.009705	3.546252	2.420328
Damby et al.	Submerged: A549	DQ12	IL-6	64.94	3.01134	2.176746	1.690412
	Submerged: dTHP-1		IL-1β	45.91	5	4.624336	3.099606
Kurtz-Chalot et al.	Submerged: RAW 246.7	DQ12	TNF- α	15.75	2.538474	1.422299	0.857813
Nattrass et al.	Submerged: J774	DQ12	TNF- α	n.d.	0.775677	0.484474	0.340345
Øya et al.	Submerged: dTHP-1	Min-U-Sil	IL-1β	76.14	1.566178	1.007269	0.68373
Pailleux et al.	Submerged: RAW 246.7	DQ12	TNF- α	21.74	4.495661	3.740628	2.229478
Rong et al.	Submerged: dTHP-1	DQ12 <1 μm	TNF- α	73.17	2.922007	2.025504	1.446472
			IL-6	105.26	4.732394	4.299903	3.300024
			IL-1β	23.76	0.559717	0.396936	0.310921
		DQ12 3-5 μm	TNF- α	48.72	1.657491	0.948116	0.617497
			IL-6	73.08	5.432732	4.47286	3.403692
			IL-1β	17.91	0.435729	0.345365	0.265956
Scheel et al.	Submerged: RAW 246.7	DQ12	TNF- α	13.12	1.910378	1.295223	1.084126
Singh et al.	Submerged: A549	DQ12	IL-8	n.d.	0.613503	0.492834	0.431116
Combined α-quartz	Rat inhalation	Min-U-Sil	PMN influx	n.d. – 92.62			0.168972
Cappellini et al.	Submerged: A549/dTHP-1	CeO_2 NM212	IL-1β	42.23	1.096177		
			IL-6	12.23	0.511335		
Kim et al.	Submerged: MH-S	CeO_2 (23)	IL-6	43.30	1.107064		
		CeO_2 (88)	IL-6	43.30	0.982811		
Combined nano-CeO_2	Rat inhalation	CeO_2	PMN influx	n.d. – 99.08			0.389696

n.d. = not determined, typically due to very low error in graphs therefore no distinction between average and error bar using software.

A magnitude change of <1 (i.e. less than one factor of 10 difference between BMDL and BMDU) indicates good quality data and is highlighted in green text.

Table S4 Model comparisons for α-quartz using log-log regression analysis

Criterion of comparability	Cell types	Endpoint (number of comparisons with dose-dependent response associations (CIs > 1))			
		**IL-8**	**IL-6**	**IL-1β**	**TNF- α**
Between all cells of the same phenotypes	Epithelial cell, submerged	1/2 comparisons suggest dose-response (Singh, 2007) [9]	1/1 comparison suggests dose-response (Damby, 2016) [7]	N/A	N/A
	Macrophage, submerged	N/A	1/4 comparisons suggest dose-response (Rong, 2013) [12]	1/4 comparisons suggest dose-response (Øya, 2019) [13]	4/9 comparisons suggest dose-response, with overlap between Boyles 2018 (J774A1 cells) [20] and Balduzzi 2004 [11] (*P*=.393) and Balduzzi 2004 and Nattrass 2017 [17] (*P*=.113)
	Epithelial cell, ALI	N/A	N/A	N/A	N/A
	Co-culture, ALI	1/2 comparisons suggest dose-response (Barasova, 2020a) [1]	1/2 comparisons suggest dose-response (Barasova, 2020a) [1]	0/2 comparisons with dose-response.	0/2 comparisons with dose-response.
Between specific cell lines	Epithelial cell, submerged: A549	1/2 comparisons suggest dose-response (Singh, 2007) [9]	1/1 comparison suggests dose-response (Damby, 2016) [7]	N/A	N/A
	ALI: Primary Human Bronchial Epithelial cells	N/A	N/A	N/A	N/A
	Macrophage, submerged: dTHP-1	N/A	1/3 comparisons suggest dose-response (Rong, 2013) [12]	1/2 comparisons suggest dose-response (Øya, 2019) [13]	1/3 comparisons suggest dose-response. (Rong, 2013) [12]
	Macrophage, submerged: RAW 246.7	N/A	N/A	N/A	2/5 comparisons suggest dose-response, but no overlap (*P*<.001)
	Macrophage, submerged: J774	N/A	N/A	N/A	2/2 comparisons suggest dose-response, but no overlap (*P*<.001)
	Macrophage, submerged: NR8383	N/A	N/A	N/A	Only 1 comparison (no response). (Albrecht 2009) [14]

Table S5 Model comparisons for nano-CeO_2 using log-log regression analysis

Criterion of comparability	Cell types	Endpoint (number of comparisons with dose response associations (CIs >1))			
		IL-8	IL-6	IL-1β	TNF-α
Between all cells of the same phenotypes	Co-culture	0/1	1/2 Cappellini, 2020 [24]	1/2 Cappellini, 2020 [24]	0/2
	Macrophage, submerged	N/A	2/2 Kim, 2014	N/A	N/A
	Co-culture, ALI	N/A	0/1	0/1	0/1
Between specific cell lines	ALI/Co-culture): A549+dTHP-1	0/1	1/3 Cappellini, 2020 [24]	1/3 Cappellini, 2020 [24]	0/3
	Macrophage, submerged: MH-S	N/A	2/2 Kim, 2014 [28]	N/A	N/A

References

1. Barosova, H., et al., Use of EpiAlveolar lung model to predict fibrotic potential of multiwalled carbon nanotubes. ACS nano, 2020. 14(4): p. 3941-3956.

2. Chortarea, S., et al., Repeated exposure to carbon nanotube-based aerosols does not affect the functional properties of a 3D human epithelial airway model. Nanotoxicology, 2015. 9(8): p. 983-993.

3. Chortarea, S., et al., Human asthmatic bronchial cells are more susceptible to subchronic repeated exposures of aerosolized carbon nanotubes at occupationally relevant doses than healthy cells. ACS nano, 2017. 11(8): p. 7615-7625.

4. Chortarea, S., et al., Profibrotic activity of Multiwalled carbon nanotubes upon prolonged exposures in different human lung cell types. Applied In Vitro Toxicology, 2019. 5(1): p. 47-61.

5. Wottrich, R., S. Diabaté, and H.F. Krug, Biological effects of ultrafine model particles in human macrophages and epithelial cells in mono-and co-culture. International journal of hygiene and environmental health, 2004. 207(4): p. 353-361.

6. Herseth, J.I., et al., Role of IL-1β and COX2 in silica-induced IL-6 release and loss of pneumocytes in co-cultures. Toxicology in Vitro, 2009. 23(7): p. 1342-1353.

7. Damby, D.E., et al., The in vitro respiratory toxicity of cristobalite-bearing volcanic ash. Environmental research, 2016. 145: p. 74-84.

8. Schins, R.P., et al., Persistent depletion of I kappa B alpha and interleukin-8 expression in human pulmonary epithelial cells exposed to quartz particles. Toxicology and applied pharmacology, 2000. 167(2): p. 107-117.

9. Singh, S., et al., Endocytosis, oxidative stress and IL-8 expression in human lung epithelial cells upon treatment with fine and ultrafine TiO2: role of the specific surface area and of surface methylation of the particles. Toxicology and applied pharmacology, 2007. 222(2): p. 141-151.

10. Tarantini, A., et al., Toxicity, genotoxicity and proinflammatory effects of amorphous nanosilica in the human intestinal Caco-2 cell line. Toxicology in Vitro, 2015. 29(2): p. 398-407.

11. Balduzzi, M., et al., In vitro effects on macrophages induced by noncytotoxic doses of silica particles possibly relevant to ambient exposure. Environmental research, 2004. 96(1): p. 62-71.

12. Rong, Y., et al., Particle-size-dependent cytokine responses and cell damage induced by silica particles and macrophages-derived mediators in endothelial cell. Environmental toxicology and pharmacology, 2013. 36(3): p. 921-928.

13. Øya, E., et al., Hyphae fragments from A. fumigatus sensitize lung cells to silica particles (Min-U-Sil): increased release of IL-1β. Toxicology in Vitro, 2019. 55: p. 1-10.

14. Albrecht, C., et al., Evaluation of cytotoxic effects and oxidative stress with hydroxyapatite dispersions of different physicochemical properties in rat NR8383 cells and primary macrophages. Toxicology in vitro, 2009. 23(3): p. 520-530.

15. Boudard, D., et al., In vitro cellular responses to silicon carbide particles manufactured through the Acheson process: impact of physico-chemical features on pro-inflammatory and pro-oxidative effects. Toxicology in Vitro, 2014. 28(5): p. 856-865.

16. Kurtz-Chalot, A., et al., Impact of silica nanoparticle surface chemistry on protein corona formation and consequential interactions with biological cells. Materials Science and Engineering: C, 2017. 75: p. 16-24.

17. Nattrass, C., et al., The effect of aluminium and sodium impurities on the in vitro toxicity and pro-inflammatory potential of cristobalite. Environmental Research, 2017. 159: p. 164-175.

18. Pailleux, M., et al., New insight into artifactual phenomena during in vitro toxicity assessment of engineered nanoparticles: Study of TNF-α adsorption on alumina oxide nanoparticle. Toxicology in Vitro, 2013. 27(3): p. 1049-1056.

19. Scheel, J., et al., Exposure of the murine RAW 264.7 macrophage cell line to hydroxyapatite dispersions of various composition and morphology: assessment of cytotoxicity, activation and stress response. Toxicology in Vitro, 2009. 23(3): p. 531-538.

20. Boyles, M.S., et al., Assessing the bioactivity of crystalline silica in heated high-temperature insulation wools. Inhalation toxicology, 2018. 30(7-8): p. 255-272.

21. Castranova, V., et al., Effect of inhaled crystalline silica in a rat model: time course of pulmonary reactions, in Oxygen/Nitrogen Radicals: Cell Injury and Disease. 2002, Springer. p. 177-184.

22. Porter, D.W., et al., Time course of pulmonary response of rats to inhalation of crystalline silica: NF-kappa B activation, inflammation, cytokine production, and damage. Inhalation toxicology, 2002. 14(4): p. 349-367.

23. Porter, D.W., et al., Progression of lung inflammation and damage in rats after cessation of silica inhalation. Toxicological Sciences, 2004. 79(2): p. 370-380.

24. Cappellini, F., et al., Dry generation of CeO2 nanoparticles and deposition onto a co-culture of A549 and THP-1 cells in air-liquid interface—dosimetry considerations and comparison to submerged exposure. Nanomaterials, 2020. 10(4): p. 618.

25. Loret, T., et al., Air-liquid interface exposure to aerosols of poorly soluble nanomaterials induces different biological activation levels compared to exposure to suspensions. Particle and Fibre Toxicology, 2016. 13(1): p. 1-21.

26. Sauer, U.G., et al., Applicability of rat precision-cut lung slices in evaluating nanomaterial cytotoxicity, apoptosis, oxidative stress, and inflammation. Toxicology and applied pharmacology, 2014. 276(1): p. 1-20.

27. Delaval, M., et al., Assessment of the oxidative potential of nanoparticles by the cytochrome c assay: assay improvement and development of a high-throughput method to predict the toxicity of nanoparticles. Archives of toxicology, 2017. 91(1): p. 163-177.

28. Kim, Y.H., et al., Comparative lung toxicity of engineered nanomaterials utilizing in vitro, ex vivo and in vivo approaches. Journal of Nanobiotechnology, 2014. 12(1): p. 1-12.

29. Keller, J., et al., Time course of lung retention and toxicity of inhaled particles: short-term exposure to nano-Ceria. Archives of toxicology, 2014. 88(11): p. 2033-2059.

30. Schwotzer, D., et al., Effects from a 90-day inhalation toxicity study with cerium oxide and barium sulfate nanoparticles in rats. Particle and fibre toxicology, 2017. 14(1): p. 1-20.

31. Barosova, H., et al., An in vitro lung system to assess the proinflammatory hazard of carbon nanotube aerosols. International journal of molecular sciences, 2020. 21(15): p. 5335.
